# Adsorption of Copper Ions to Secondary Microplastics in Seawater

**DOI:** 10.3390/molecules31162873

**Published:** 2026-08-17

**Authors:** Aneta Dorota Pacyna-Kuchta, Jakub Karczewski, Anetta Zioła-Frankowska, Lukasz Wolski, Marcin Łapiński, Kinga Kujawska, Marcin Frankowski

**Affiliations:** 1 Department of Biotechnology and Microbiology, Faculty of Chemistry, Gdańsk University of Technology, 11/12 Narutowicza Street, 80-233 Gdańsk, Poland; 2 Advanced Materials Centre, Faculty of Applied Physics and Mathematics, Gdańsk University of Technology, 11/12 Narutowicza Street, 80-233 Gdańsk, Poland; jakub.karczewski@pg.edu.pl (J.K.); marcin.lapinski@pg.edu.pl (M.Ł.);; 3 Faculty of Chemistry, Adam Mickiewicz University Poznań, Uniwersytetu Poznańskiego 8 Street, 61-614 Poznań, Poland; anetta.ziola-frankowska@amu.edu.pl (A.Z.-F.); wolski.lukasz@amu.edu.pl (L.W.); marcin.frankowski@amu.edu.pl (M.F.)

**Keywords:** single-use plastic, copper, environmental risk, adsorption, marine litter, plastic pollution

## Abstract

One of the main sources of secondary microplastics (MPs) in the marine environment is single-use plastic products. However, research on their adsorption capabilities is still limited. In this study, we used a representative set of well-characterized micro-sized fragments, films, and foam to evaluate differences in copper(II) adsorption via a series of batch adsorption experiments. We aimed to understand how the adsorption capacity of Cu(II) differs between a set of secondary MPs in model seawater. We examined the effect of particle size, surface hydrophobicity, and salinity as factors influencing adsorption. The highest adsorption capacity was observed for foam fragments made from a clamshell PS food container followed by a food tray made from PP (591 ± 168 and 353 ± 45 µg/g of MP, respectively). The presence of a higher salinity environment had no negative effect on the adsorption capacity, except that of spherical PS. Our results suggest that the chosen MPs (hard fragments and films) do not have a high ability for Cu(II) adsorption, except for expanded PS and PP films. This study also highlights the difficulties associated with using irregular pieces of post-consumer plastic in model experiments.

## 1. Introduction

Microplastics (MPs) are one of many pollutants entering the marine environment that are causing increasing concern. Depending on their origin, they can be divided into primary MPs—which are produced in microplastic form (e.g., beads used in cosmetics)—and secondary MPs, which are formed as a result of the fragmentation of larger plastic items. Single-use plastic products (SUPs) are especially problematic in the context of environmental pollution, as they are only used once and then thrown away. Some, like plastic takeaway food containers, can easily release MPs of irregular shapes and organic additives during daily use, e.g., by storing hot food or microwave treatments [1] or by degradation in the environment, creating secondary MPs [2]. SUPs are more likely to end up in the sea than reusable options, and they represent 50% of all marine litter in the European Union (EU) [3]. To help reduce the volume and impact of certain plastic products in recent years in the EU, regulations have been introduced, for example, the ‘Directive on single-use plastics’ [3]. However, as global plastic production is expected to increase, the amount of plastic entering the ecosystems is also expected to increase accordingly.

During fragmentation, plastics break down into pieces of varying sizes, including pieces ranging from 5 to 25 mm in size (known as mesoplastics) and MPs with a diameter of less than 5 mm [4]. A growing number of studies are focusing on the impact that MPs may have on ecosystems and humans, including the transfer and chemical bioavailability of pollutants adsorbed on their surface into the food chain [5]. MP particles typically have strong hydrophobic properties and a large specific surface area, and because of these characteristics, many pollutants can adsorb to their surface from contaminated environments (e.g., harbors or wastewater treatment plants). They may then act as a carrier for toxic compounds, possibly constituting a threat for marine organisms, by combining physical stress and chemical interactions [6,7]. Experimental adsorption studies on metals usually use different types of pure polymer in the form of spherical microbeads or pellets, either raw or artificially aged [2] (Supplementary Materials, Table S1). Secondary MPs, if used, are often ground down into fine particles to ensure a more uniform particle size distribution. Larger pieces of plastic of irregular shapes originating from SUPs are not well represented in the data, even though they are commonly found in the environment and may consequently be ingested by marine creatures [8,9]. They usually have a heterogeneous composition and contain additional property-enhancing components, including anti-static agents, plasticizers, and dyes, which can affect adsorption in the aquatic environment [2]. The density of post-consumer products may also differ from the values given in raw material databases, due to the presence of additives, polymer contamination arising during production or recycling, and the effects of weathering [10].

Copper ions and MPs, as well as bigger plastic fragments, often co-exist in the aquatic environment, which leads to interactions between them that may further affect their environmental fate, bioavailability, and toxicity [6,7]. Harbors and bays are particularly vulnerable to anthropogenic pressure, including metal leaching of Cu from the use of antifouling paints containing copper-based biocidal pigments and ship scrubbers [11]. The combined effect of MPs with adsorbed Cu may have, e.g., a negative health impact on the survival and growth of fish [12]. Thus, understanding the behavior and mechanism of metal adsorption in relation to bigger size secondary MPs is important for the assessment of their environmental fate.

Trace elements can be found in water in chemical forms that may have different mobility and bioavailability, affected by environmental conditions and interactions with microorganisms. In surface water, copper can be found mainly as the cupric form, Cu(II), which can form stable complexes with, e.g., -NH_2_, -SH, and -OH functional groups of dissolved organic matter [13]. Cu is also stored and can be released from sediments as it mainly exists in potentially available forms [14]. Compound adsorption to MPs is a complicated process and is affected by many variables, including surface physicochemical properties of plastic and water characteristics such as pH and salinity, as well as the presence of organic matter and other ions [7,15,16]. Particle size, shape and density—which determine plastic buoyancy– are also crucial for environmental pathways [10]. External factors such as solar irradiation can lead to the formation of new functional groups on the plastic’s surface [17]. MPs’ external morphological characteristics and their properties may also change through abrasion caused by wave action, or contact with sand or other objects in the water [18]. Thus, even buoyant MPs may sink with time, due to hole and biofilm creation on their surface [18], which also change how compounds are adsorbed to MPs’ surfaces, enhancing the adsorption and stabilization of ions, including Cu(II) [5,19].

Spherical microspheres or pellets are useful for gaining a better understanding of the general mechanism of adsorption; however, there are significant differences between these and the particle fragments derived from consumer products found in the environment. This is particularly important given that the same type of polymer can be produced in different forms, depending on its intended use. For example, foamed polystyrene used in the consumer, packaging, construction, and marine sectors may be either expanded (EPS) or extruded (XPS). Differences during the production process between EPS and XPS result in structural differences between them [20], which may then result in different adsorption capabilities. In studies on adsorption, these differences are often not taken into account.

**Figure 1:**
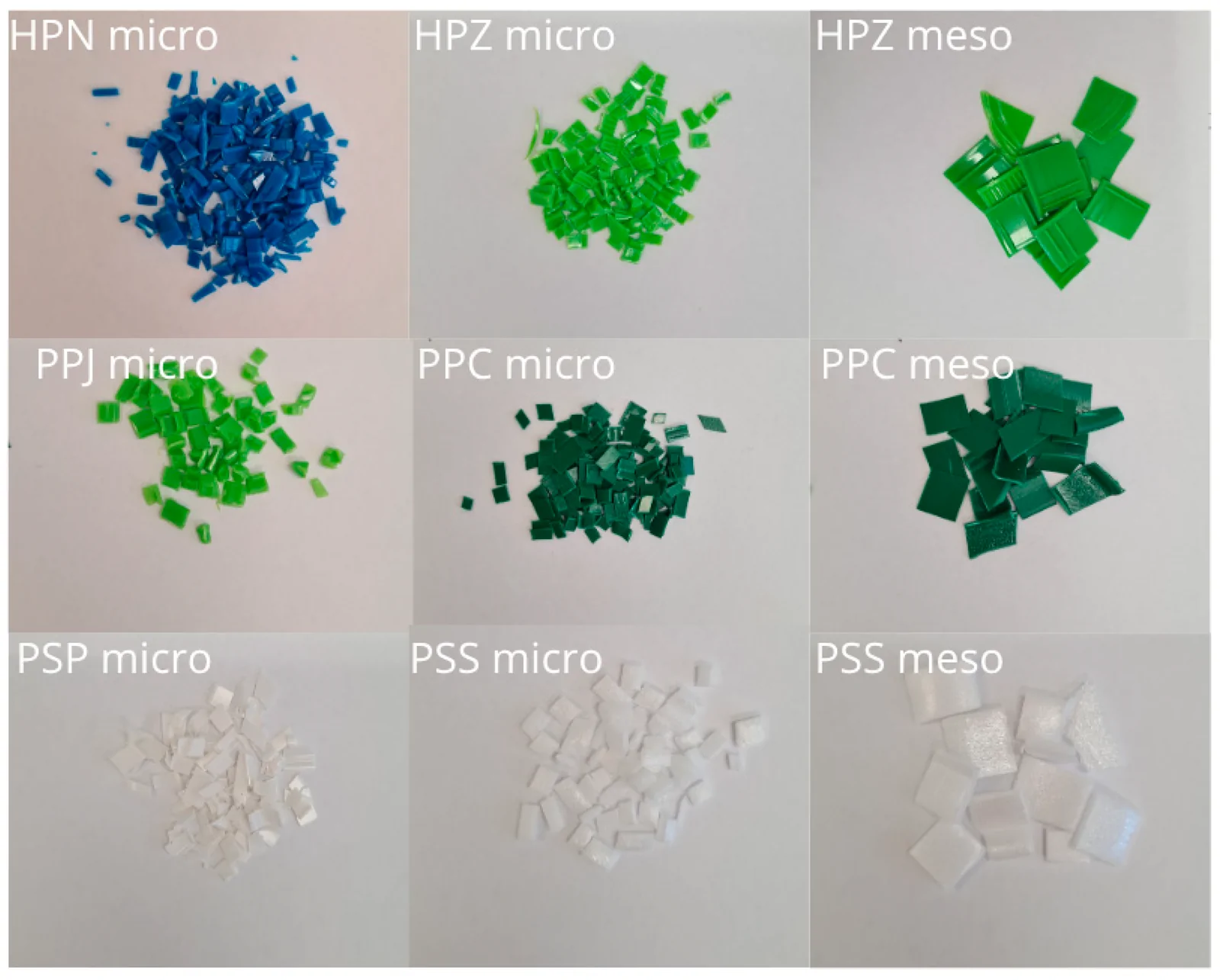
Post-consumer plastics item form. Abbreviations: HPN—blue bottle cups, HDPE; HPZ—green bottle cups, HDPE; PPJ—green bottle cups, PP; PPC—green food tray, PP; PSP—white coffee cup lid, PS; PSS—white food tray, PS.

Our main aim was to gain a better understanding of how effective larger secondary plastics might be as a carrier for Cu(II) in a low-salinity environment. Firstly, we investigated the interfacial interactions between dissolved Cu(II) and secondary plastics to evaluate the adsorption capacity and kinetics of Cu(II) adsorption on plastics depending on the type, form, and size fraction (analyzing micro- and mesoplastics). We used the three most commonly found plastic types in the marine environment (polypropylene PP, high-density polyethylene HDPE, and polystyrene PS), made from six post-consumer SUPs (Figure 1), with their surface properties characterized using SEM, FTIR, X-ray photoemission spectroscopy, and water contact angle. All of the chosen SUPs have already been in use, which is why they show some signs of wear; they have not been artificially aged.

The second objective was to compare MPs’ adsorption capabilities (post-consumer MPs and microspheres) in low-salinity surface waters (7‰) to the higher salinity found, e.g., in the estuaries (20‰). The analysis was conducted using environmentally relevant pH levels and artificially prepared seawater.

Additionally, we have conducted a preliminary study to compare how the type of water matrix (low-salinity model seawater and fresh Baltic low-salinity seawater) can cause possible differences in Cu(II) adsorption. Creating more environmentally relevant study conditions is necessary to better understand the process of adsorption, even though it is a challenge to fully imitate them at laboratory scale. While artificial seawater is a mixture of electrolytes in deionized water that chemically resembles the natural matrix, natural seawater additionally contains other metal ions and organic contaminants, as well as colloids and soluble particles. Thus, it is a more complex system and more difficult to fully imitate at laboratory scale. Here, we used only commercially bought microspheres in the form of pure polymers (PP, PS and PE), so that the matrix type was the only variable.

## 2. Results and Discussion

### 2.1. MPs Surface Characteristics

**Figure 2:**
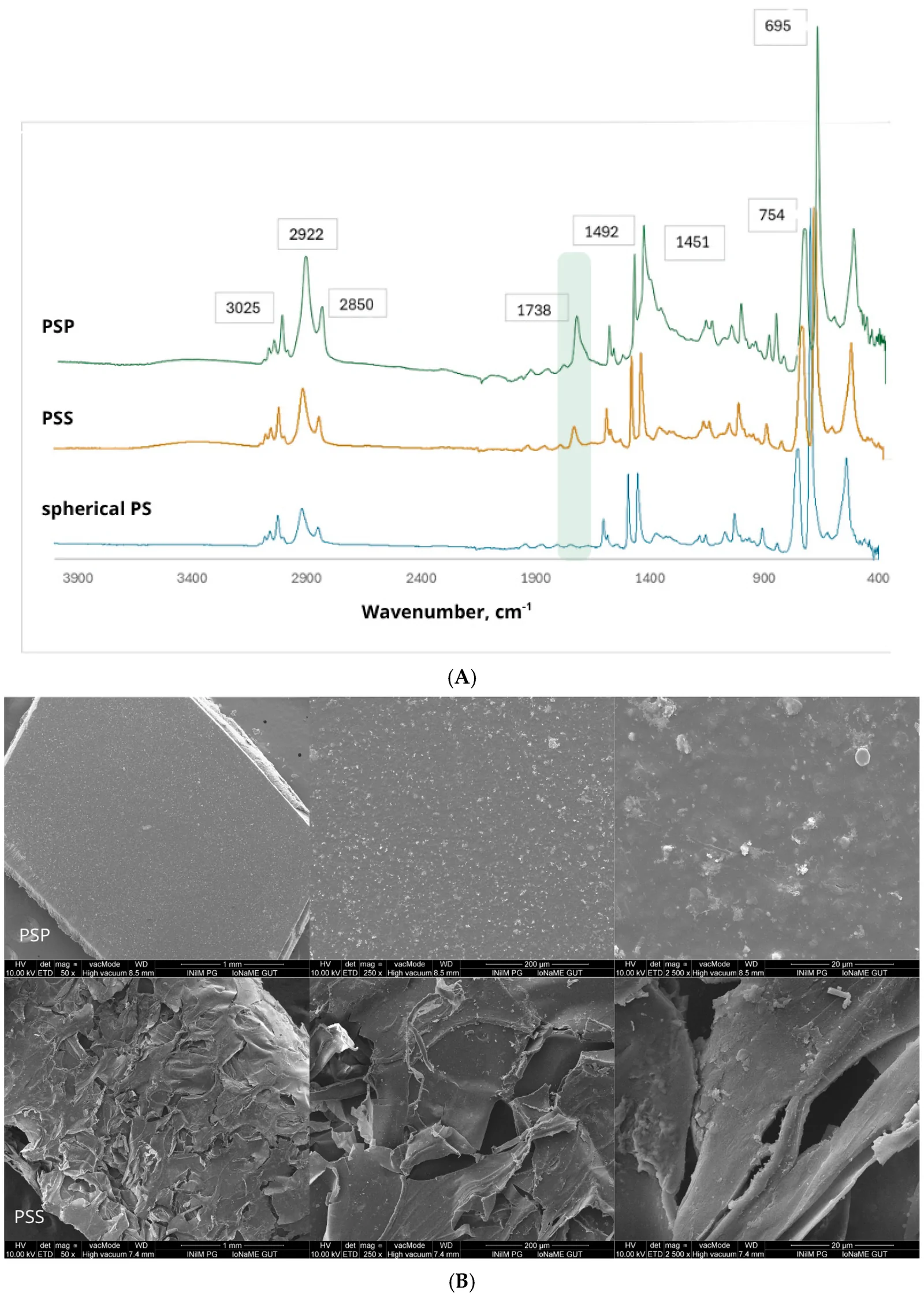
(**A**) Infrared spectra with identification of absorption peaks and functional groups. A green area shows the presence of an additional peak (1736–1738 cm^−1^), observed only in post-consumer products. (**B**) SEM morphology on secondary MPs; PSP–PS white coffee cup lid, (magnification 50×, 250× and 2500×); PSS–PS white food tray (magnification 50×, 250× and 2500×).

For our experiment, we selected two types of each kind of secondary plastic: PS (EPS foam and XPS film), HDPE (hard fragments, different colors) and PP (hard fragments and film). To gain a better understanding of the structure and surface properties of secondary plastic particles, they were compared with market-available raw microspheres. FTIR-ATR results with characteristic peaks for functional groups on each polymer were identified based on Smith [21] and Godoy et al. [15]. For PS (Figure 2A), they can be observed at wavenumber 695 cm^−1^ for aromatic ring bend, 754 cm^−1^ for aromatic C-H bend, 1492 and 1600 cm^−1^ for aromatic ring modes, 2924 and 2850 cm^−1^ for CH_2_ asymmetric and symmetric stretches, and 3025 cm^−1^ for aromatic C-H stretches. There are also weak overtone and combination bands from around 1650 to 2000 cm^−1^, called benzene fingers [21]. Additional peaks appear for both commercial products, at 1736–1738 cm^−1^.

EPS has great insulating properties, mainly due to the air trapped within and between the beads that it is made of. As PSS was made from an EPS clam-shell container used as food packaging, this structure provides better isolation for food storage; but, interparticle air in irregular gaps makes this material prone to some limited water absorption. Among all post-consumer MPs, PSS had the most cracks and irregularities on its surface, with visible air gaps in the structure (Figure 2B). Cracks can increase the surface area, thus leading to greater adsorption of metal on its surface [6]. Extruded PS structure is typically smoother and more closed, has higher density than EPS, and has no gaps or voids, thus inhibiting water absorption [20]. PSP, which was made out of an XPS coffee cup lid, had a smoother surface and fewer irregularities, with no visible air gaps. It also had the highest density and was the only plastic that sank to the bottom of the flask from the beginning of the experiment. Although our post-consumer samples were not additionally aged, they showed signs of weathering, including a build-up of small particles, which was also observed on PET, PE, and PS secondary microplastic affected by UV light and water [22,23].

**Figure 3:**
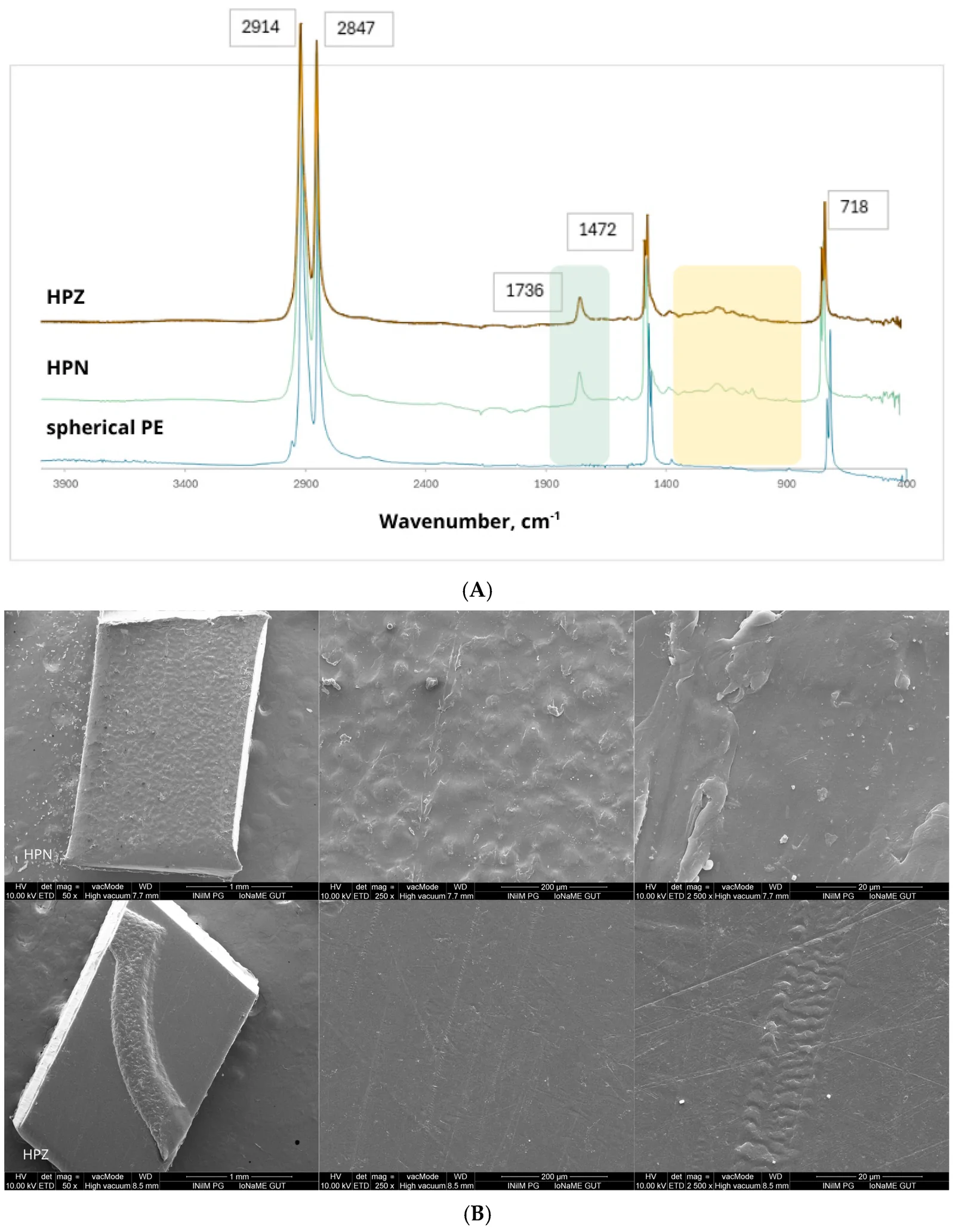
(**A**) Infrared spectra with identification of absorption peaks and functional groups. A green and yellow area shows the presence of additional peaks (1736 and between 1400 and 900 cm^−1^), observed only in post–consumer products. (**B**) SEM morphology on secondary MPs; HPN–HDPE blue bottle cups (50×, 250× and 2500×), HPZ–HDPE green bottle cups (50×, 250× and 2500×).

For PE, characteristic peaks for functional groups can be observed at wavenumbers 718 and 730 cm^−1^ for CH_2_ splitting, 1472 cm^−1^ for C-H link, 2847 cm^−1^ for CH_2_ symmetric C-H stretch, and 2914 cm^−1^ for CH_2_ asymmetric C-H stretch. For post-consumer products, additional peaks appear at 1736 and between 1400 and 900 cm^−1^ (Figure 3A).

PE is one of the most widely used synthetic polymers worldwide, and for this reason it is also often found in the marine environment [17]. Bottle cups are typically made of HDPE or PP and often contain additives to improve their properties, such as slip agents, anti-statics, colors, and pigments. Both HPN and HPZ, which were made of HDPE, had some irregularities on the surface, caused by the natural use of the product (Figure 3B). HPZ had more scratches, while HPN had small particles emerging on the surface.

**Figure 4:**
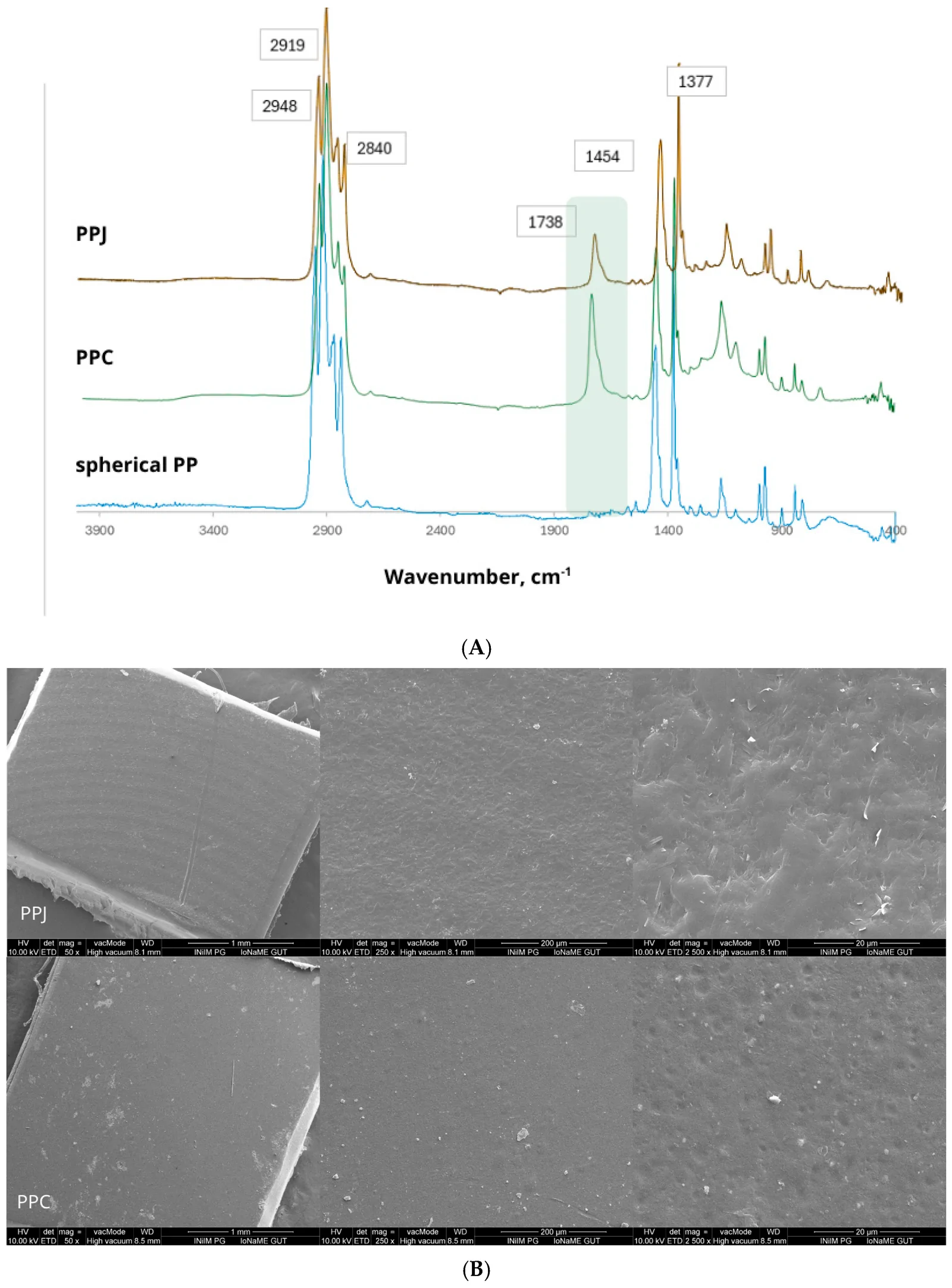
(**A**) Infrared spectra with identification of absorption peaks and functional groups. A green area shows the presence of an additional peak (1738 cm^−1^), observed only in post-consumer products. (**B**) SEM morphology on secondary MPs; PPJ–PP green bottle cups (50×, 250× and 2500×), PPC–PP green food tray (50×, 250× and 2500×).

PP is commonly used for packaging due to its low cost, high tensile strength, and versatility [17]. The characteristic peaks appear at 1377 cm^−1^ for CH_3_ deformation, 1455 cm^−1^ for CH_2_ bend, 2840 and 2919 cm^−1^ for CH_2_ asymmetric and symmetric stretches, and 2950 cm^−1^ for CH_3_ stretches. For post-consumer products, additional peaks also appear at 1738 cm^−1^ (Figure 4A). PPC had a smooth surface with small pits, and particle build-up, while PPJ had more irregularities (Figure 4B).

Microspheres used for comparison had all spherical shapes, but varied in size. The smallest were PS microspheres, with a mean size of 9.5–11.5 µm. They had a smooth surface with flakes starting to show up after the adsorption experiment, probably due to constant movement in the water (Supplementary Materials, Figure S1). The PE microsphere surface was smoother than PP, but also with some irregularities and a wrinkled structure. PP microspheres were the largest spherical MPs, with a diameter of 2.45 ± 0.05 mm, and were comparable in size to the post-consumer microplastics used in this experiment. Under magnification, they had a rough surface with cracks.

**Figure 5:**
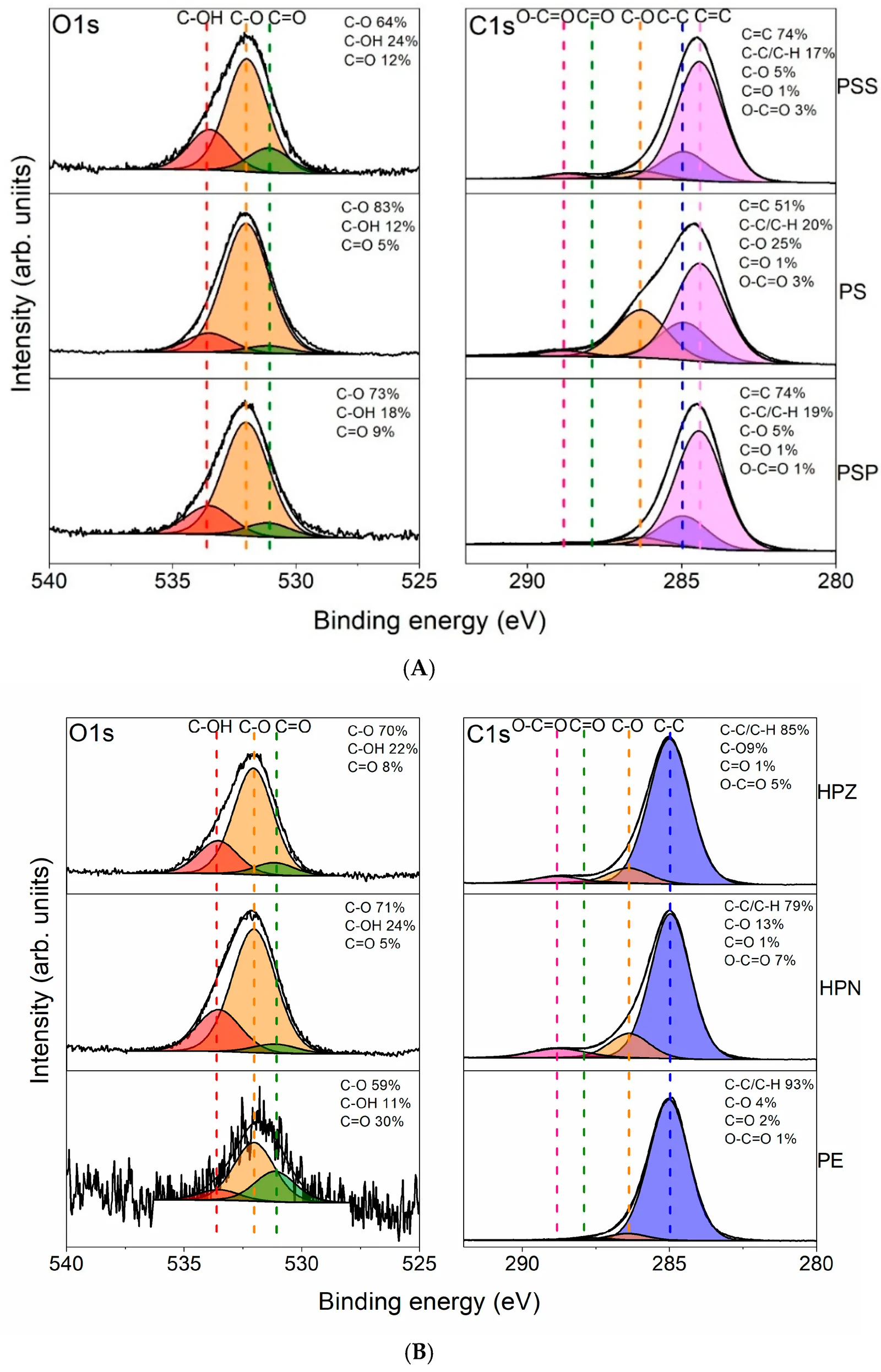

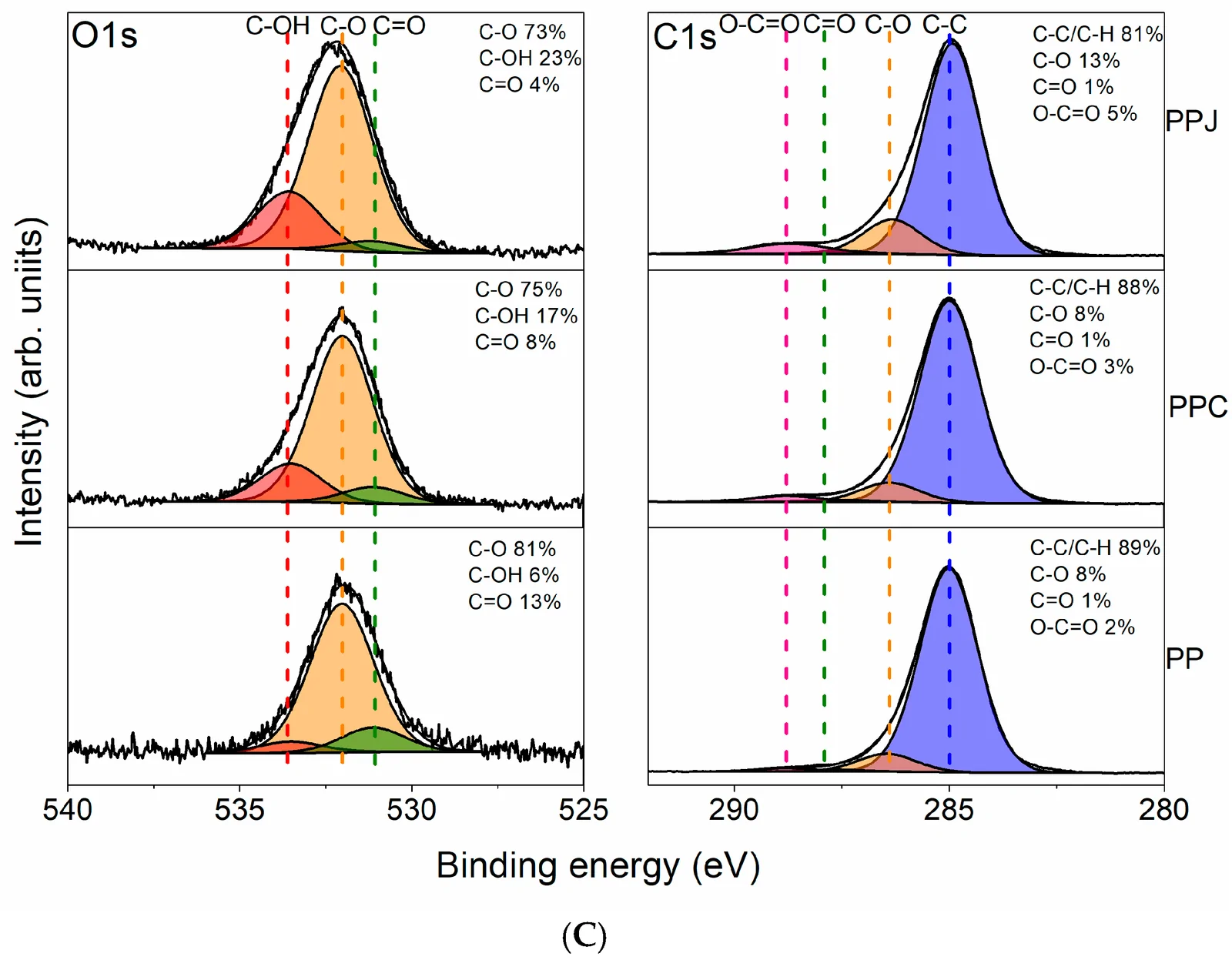
High-resolution X-ray photoemission spectroscopy spectra of the O1s and C1s regions recorded for three polystyrene (**A**), polyethylene (**B**), and polypropylene (**C**) types.

The presence of different functional groups may affect adsorption capacity, as it depends on intermolecular interactions between the pollutants and the polymer, such as van der Waals interactions, hydrogen bonding interactions, and cavity formation [24]. Therefore, the differences in the chemical structure of the polymers may impact the adsorption and the possible interactions between MPs and metals [15]. All post-consumer products used in the experiment were created and used for food contact. The additional peaks present only on them can be an indication of surface-bound additives [25]. Typical additions to bottle cap plastic include processing and thermal stabilizers such as phosphites, antioxidants for providing stabilization during service life, UV light stabilizers, slip agents, and anti-statics [25]. Some chemical additives such as catalysts, fillers, and plasticizers may generate active sites for the sorption process [26]. However, the identification of an exact additive may be difficult, as they can exist under different crystalline forms with a specific IR signature, and multiple antioxidants can be added to one product [27,28]. For example, a phenolic antioxidant, Irganox 1076^®^ (Ludwigshafen, Germany) (octadecyl-3,5-di-tert-butyl-4-hydroxyhydrocinnamate), is commonly added to food packaging materials. In its crystalline form, the characteristic IR absorption bands of the phenol and ester functions were observed at 3639 cm^−1^ and 1733 cm^−1^, respectively [29]. However, in a dispersed state, the bands of phenol and ester functions were shifted even up to 3651 cm^−1^ and 1742 cm^−1^, respectively [29]. Some studies identified the characteristic absorption peak of carbonyl structure at precisely 1738 cm^−1^ [30]. For Irganox 1010^®^, the characteristic carbonyl absorption peak in PE was determined to be 1739 cm^−1^ [31] and 1744 cm^−1^ in PP [32]. Alternatively, these peaks could be a sign of plastic aging due to its use, as peaks between 1734 and 1736 cm^−1^ observed for eroded PE and oxidized HDPE were identified as ester carbonyl (-COO-) and C=O stretching (Ref. [33] and references therein; Ref. [34]). However, additives can also be lost or degraded over time, which can lead to shifts in absorbance peaks [35]. Therefore, the separate determination of additives and polymer aging products is a very difficult task [35]. To better understand the surface properties of the samples, the O1s and C1s X-ray photoemission spectroscopy spectra were analyzed (Figure 5). The O1s spectra were deconvoluted into three characteristic peaks corresponding to C=O, C–O, and C–OH functional groups. The main difference observed between raw spherical MPs and secondary MPs was related to the relative intensity of hydroxyl (–OH) groups, which was higher in post-consumer MPs (17–24% vs. 6–12% in spherical MPs), and the C=O component, which was more pronounced in spherical MPs, except polystyrene samples (Figure 5). The C1s spectra were deconvoluted into four components corresponding to C–C, C–O, C=O, and O–C=O bonds. Additionally, in the case of polystyrene, the dominant C=C component was included in the fitting procedure. Differences in the relative intensities of the O–C=O and C–O peaks observed in the C1s spectra may indicate surface oxidation (Figure 5). The oxygen atoms present in the spherical raw PS may result from oxidation during processing or transportation, as previously seen in other studies [36].

In the case of BET measurements, we were only able to determine the specific surface area values for the PE and PS microspheres (see commentary in the Supplementary Materials, Text S1). The nitrogen adsorption isotherm for spherical PE, measured at 77 K, showed low but clearly positive adsorption across the entire range of relative pressures. The isotherm curve was smooth and free of non-physical artifacts, such as negative adsorption volumes or instabilities in the low P/P_0_ range. BET analysis performed in the standard range of P/P_0_ = 0.05–0.30 led to a very good linearity of fit (correlation coefficient r ≈ 0.9996), confirming the formal correctness of the BET model in this range. The determined specific surface area was 0.163 m^2^/g, which is a very low value, but realistic for powdered polymer materials with limited porosity. Such a low specific surface area is close to the lower limit of sensitivity of the nitrogen adsorption method at liquid nitrogen temperature. Nevertheless, the data obtained were consistent and free from systematic errors observed for samples with even lower adsorption capacity. For spherical PS, the specific surface area was 0.301 m^2^/g; however, the isotherm had an atypical shape and was slightly ‘open’, which may suggest that the material does not have a micro- and mesopore structure accessible to nitrogen.

### 2.2. Adsorption in Artificial Seawater

#### 2.2.1. General Overview

The adsorption capacity may vary based on the different chemical structures of each polymer [15] and the possible direct interactions between metals and plastic in a liquid medium [37,38]. Secondary microplastics from everyday products are more challenging than raw spherical MPs or pellets because they not only come in a variety of forms, shapes and sizes, but also contain additives that are difficult to identify and which vary depending on the product’s intended use. Irregular, rough surfaces and the increased oxygen-containing groups on the surface of the MPs that may form complexes with the metal may enhance adsorption [36]. All secondary MPs showed an increase in hydroxyl (–OH) groups, compared to pure polymers, suggesting that they might be contributing to the increased adsorption capacity.

Analysis using a linear mixed model revealed a significant effect of material (*p* < 0.001), time (*p* < 0.001), and the material × time interaction (*p* < 0.001) on Cu(II) adsorption. The highest adsorption values were obtained for the PSS material, which reached an average of 591 µg/g after 28 h. Tukey’s post hoc tests showed that adsorption on PSS was significantly higher than for HPN (*p* = 0.00047), HPZ (*p* = 0.00050), HPZ-meso (*p* = 0.00014), PPJ (*p* = 0.00072), PSP (*p* = 0.00030), PPC-meso (*p* = 0.0103), and PSS-meso (*p* = 0.00022). Only when compared with PPC were there no statistically significant differences (Tukey, *p* = 0.21). Statistical differences were also observed for PPC vs. PSP (*p* = 0.043), PSS-meso (*p* = 0.0296) and HPZ-meso (*p* = 0.017).

Interestingly, all MPs made from bottle cups had comparable results (PPJ, HPZ, HPN; mean ± SE 136 ± 20; 123 ± 15; 121 ± 21 µg/g, respectively), suggesting that this type of hard plastic does not have a very high adsorption capacity for Cu(II). Antistatic agents used in caps reduce static charge and dust attraction; agents such as erucamide or oleamide, which are slip-promoting additives, can be commonly found in both types [25]. They can affect adsorption, but their impact is not well studied.

Based on previous studies, the main interactions between Cu(II) ions and MPs in deionized water were identified as electrostatic interactions and surface complexation, mediated by the presence of hydroxyl and carboxyl functional groups—which carry a negative charge [16,37]—and chemisorption [15]. In studies where artificial seawater or natural media were used, authors identified direct adsorption of cations or complexes onto charged sites or neutral regions of the MPs’ surface [6], physical adsorption [7], chemisorption [39], and electrostatic and coordination bonding [40]. In addition to the many factors recognized as influencing the adsorption process (Supplementary Materials, Table S1), aging (both artificial and natural) introduces additional factors, primarily related to changes occurring on the polymer surface, such as the formation of new functional groups and peroxy radicals [36].

Adsorption is not influenced by a single characteristic, but rather by a combination of factors. Thus, the results for individual metals obtained in different studies may vary considerably. For a similar type of product used in the present study (irregular fragments of PP and PS originating from fruit boxes and an egg cup, respectively), but a different matrix (deionized water, 14 days), adsorption reached around 200–250 µg/g [15]. The maximum adsorption amounts for nylon MPs (PA6) were 146 µg/g [41] to 1060 µg/g for MPs made from used nylon rope [37]. Maximum adsorption capabilities on CPE, PVC, and PE were between 56 and 431 µg/g in Zou et al. [16] study. In a study by Han et al. [42], adsorption capacity was strictly dependent on particle size and reached 0.3–0.55 µg/g for PE and 0.1–0.2 µg/g for PP crushed pellets. Maximum adsorption capacity for aged PS and pristine PS was equal to 489 and 365 µg/g in Huang et al. [43] study.

#### 2.2.2. Particle Size Effect and Hydrophobicity

Particle size may have an effect on adsorption capacity, as all mesoplastics had lower adsorption capacity compared to MP particles, although this difference was statistically significant only in the case of PSS foam. MPs with smaller particle sizes are expected to have a higher adsorption capacity compared with particles with a bigger particle size due to the larger specific surface area [7]. Considering all types, PPC mesoplastics had a higher adsorption capacity than micro-PPJ, HPZ, HPN and PSP, but the differences were not significant (*p* > 0.894) (Table 1). In general, the adsorption capacity of Cu(II) on secondary plastic followed the order of PSS micro > PPC micro > PPC meso > PPJ > HPZ micro~HPN > PSP > PSS meso > HPZ meso. In the case of PSS, although we were unable to determine its specific surface area, this material had the highest number of cracks and air pockets in its structure; consequently, the contact area was larger than in the case of hard plastics. It was also characterized by a high standard error, which may have been due to the spatial heterogeneity of the surface and irregularities.

Based on their molecular structure, all three types of secondary plastic (PP, PS, HDPE) are highly hydrophobic [2]. However, this property may, in certain cases, be modified by the manufacturer, depending on the final product design. Also, for used, aged plastic, an increase in hydrophilicity was observed [37]. A large water contact angle (above 90°) indicates that the material is hydrophobic, whereas materials with a contact angle of less than 90° are more wettable [44]. Water contact angles showed differences in hydrophobicity between polymer types. The highest contact angle was found for polypropylene MPs (PPJ 107.8 ± 2.5°) and the lowest for polyethylene bottle cups (HPZ 85.1 ± 2.2° and HPN 87.4 ± 4.9°). The outer side of the PPC tray, as well as the PSS tray, was more hydrophobic than the inside (Table 1), which may have been deliberately altered during the manufacturing process. As PP is generally highly hydrophobic, some anti-fog or wetting agents might be blended into it to improve wettability and prevent fogging [45]. In the case of PS foam, the main reason for the improved wettability on the inner surface of the tray alone is usually the increased ability of the foam to absorb liquids that leak from the product, such as meat [46]. The differences in hydrophobicity do not seem to be high enough to have a direct high impact, as for the same type of product (bottle cups) made of more hydrophobic PP and less hydrophobic HDPE (PPJ, HPZ and HPN)—adsorption capacity was not significantly different. However, we cannot be certain that the change in hydrophobicity was insignificant for the adsorption process in the case of plastic originating from food trays (PSS and PPC).

#### 2.2.3. Effect of Salinity

**Figure 6:**
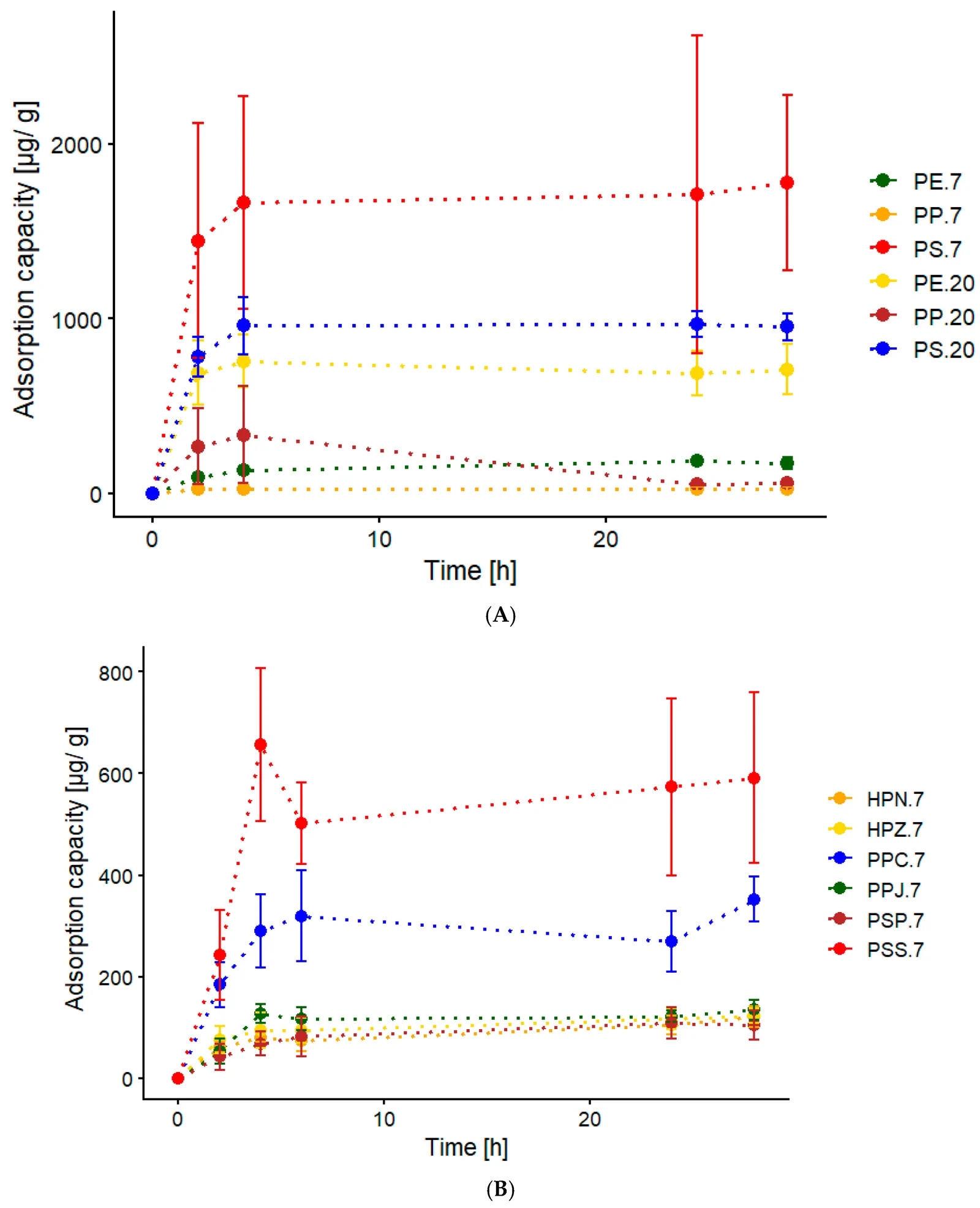

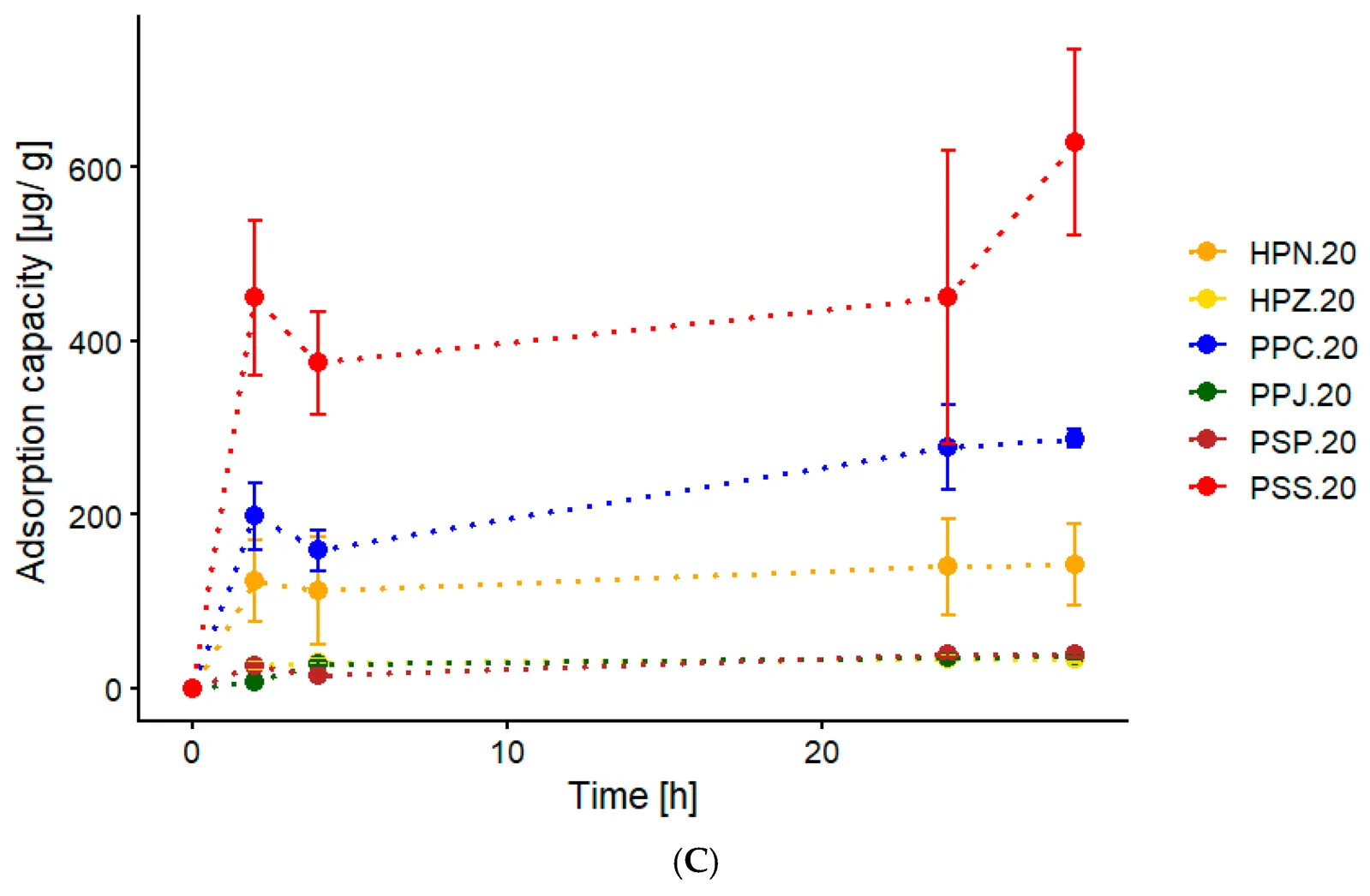
Mean adsorption capacity for spherical PP, PS and PE for salinity of 7‰ and 20‰ (**A**), post-consumer MPs for salinity of 7‰ (**B**) and 20‰ (**C**), respectively. Values are mean± SE.

Salinity is one of the key factors that can influence the adsorption properties of microplastic particles; however, its effect does not show a consistent pattern. The combined effect of salinity and the chemical composition of seawater can either increase or decrease the adsorption of metals onto polymers [15]. In this study, we analyzed the effect of salinity on the kinetics of adsorption capacity, examining both low and higher salinity levels. We investigated the kinetics of secondary microplastic particles—and, in addition, spherical microspheres—separately (Figure 6).

For secondary MPs, a linear mixed-effects model revealed significant effects of material type, incubation time, and their interactions with salinity. While no overall effect of salinity was observed (*p* = 0.238), significant salinity × time (*p* < 0.001) and material × salinity × time interactions (*p* = 0.003) indicated that the influence of salinity depended on both material type and incubation time. Post hoc comparisons showed significant differences between salinity treatments only for the PSS material at 2 h (*p* = 0.013) and 4 h (*p* = 0.001). No significant differences between salinity levels were observed for any material after 24 or 28 h of incubation. These results suggest that salinity primarily affected adsorption kinetics rather than the final adsorption capacity. Unlike spherical MP particles, some secondary MP particles (PSS in higher salinity) did not reach equilibrium after a specified period of time.

Salinity also did not exert a significant overall effect on Cu adsorption to spherical MPs; however, its influence depended on polymer type. For both spherical PP and PE, higher salinity resulted in enhanced mean adsorption (from 27.2 to 60.3 µg/g for PP and from 174 to 710 µg/g for PE). However, the mixed-effects model did not detect a significant salinity effect for PE at any individual sampling time after correction for multiple testing (*p* > 0.05). Analysis restricted to the equilibrium stage (28 h) revealed significantly higher Cu adsorption at 20 ‰ than at 7 ‰ (*p* = 0.046). This suggests a modest salinity effect that becomes apparent at the end of the experiment but is not sufficiently strong to remain significant in the full kinetic model. No significant salinity effect was detected for PP.

The opposite pattern was observed for spherical PS, which showed significantly higher adsorption at 7‰ (*p* = 0.0058). The decrease in adsorption with increased salinity could be caused by the competition between cations for active adsorption sites [15,40]. As salinity increases, the concentration of many ions also increases, including Na(I), Ca(II) and Mg(II), leading to an electrolyte ion competition effect and possibly inhibiting the electrostatic interaction between Cu(II) and MPs [7]. The increase in anion concentration, such as Cl^−^, may change metal species distribution in solution. As some positive divalent metals can be converted to M-Cl_2_ and negatively charged chlorides, this may lead to a lower activity of free metal ions and thus the adsorption may decrease significantly [37].

A pH of 7, which we used in our experiments, is an optimal solution condition for Cu(II) experiments [43]. The metal oxides tend to form precipitates in solution at pH > 8, which may decrease the adsorption capacity of MPs [7,41]. However, it is not only pH which may cause ion precipitation. In high salinity, Cu may react and form precipitates with carbonates, chlorides, sulphates, phosphates, hydroxides, and oxides, and thus result in forming, e.g., copper hydroxycarbonate and dicopper trihydroxide chloride [15,47]. Here, after 4 h, we observed precipitation in all samples where a higher salinity was used (none was observed for 7‰). Other metals, such as Cr or Pb, may also be prone to precipitation, particularly when a medium other than deionized water is used, such as wastewater [15].

The presence of electrolytes in the solution, such as Ca^2+^ from CaCl_2_ and Mg^2+^ from MgCl_2_, may also cause particle aggregation. As the divalent ions cause higher electrical double layer compression than monovalent Na^+^, the electrostatic interaction between the polymers may be changed, causing the aggregation of MPs [48,49]. This can further lead to the reduction in specific surface area and decrease in adsorption capacity [7]. Aggregation determines the mobility, distribution, and bioavailability of particles, making it one of the most important environmental behaviors of MPs. Many factors may additionally impact the aggregation of MPs, including their size and composition, electrolyte concentration, pH, and the presence of organic matter, surfactants, and light [50]. In our study, while aggregation was observed for both spherical PS and PE, the effect observed visually and under SEM was different for both of them. PE formed aggregates only at higher salinity (20‰), while PS formed aggregates at the bottom of the vial for both 7 and 20‰. In the case of PE, the aggregation effect was less significant, probably due to the larger particle size. Under magnification, although the PE particles are close to each other, each remains separate. On the contrary, the PS particles have formed tightly packed clusters, which reduces their surface area (Supplementary Materials, Figure S2). Ca^2+^ and Mg^2+^ can lead to the formation of cation bridges between negatively charged PS particles and can affect colloidal stability and lead to aggregate deposition, and this process can be enhanced with increasing temperature and particle concentration [40,49,51]. Though the effect of aggregation is often studied on nanoplastics (e.g., [49,51,52]), micro-PS particles are also susceptible to this process, as even very low salinity may cause aggregate formation, though much smaller (Supplementary Materials, Figure S1). The electrolyte-induced aggregation may explain the decreased adsorption of Cu ions on micro-PS at higher salinity. Higher adsorption at lower salinity for PS microspheres was also reported in, for example, the studies by Liu et al. [53] and Soheilian et al. [40].

However, the effect of salinity on Cu(II) adsorption may depend on polymer type and form. The enhancement with increased salinity was seen here for PE and previously, e.g., PA, PVC and PP, and could be caused by the combination of cations onto new molecules conducive to the adsorption site on the surface of MP [15,41]. This increase may be related to the “salting out” effect, in which the presence of salt ions lowers the solubility of contaminants and promotes their hydrophobic interactions with MPs [54]. Also, the reduction in the electrostatic interaction between MPs and pollutants due to the presence of NaCl in the solution and partial neutralization of the negative charge at the adsorption site on the surface of MPs may lead to higher adsorption [55]. An enhancement of adsorption at higher salinity was also observed for some organic pollutants, such as the adsorption of triclosan on PVC [55].

### 2.3. Adsorption Kinetics

Adsorption kinetics were performed to better understand the adsorption performance of Cu(II) on different polymers with contact time. Adsorbent and adsorbate can interact in two ways: by physisorption, which is the result of attractive forces between adsorbent and adsorbate, and chemisorption, which is based on the transfer or sharing of electrons between adsorbent and adsorbate [56]. We applied three kinetic models to the experimental data, namely the pseudo-first-order (PFO) and pseudo-second-order (PSO) kinetic models, as well as the intra-particle diffusion (IPD) model. Though not perfect, the first two models are often used to reveal the rate-controlling step of the adsorption, and both are extensively used to study the kinetics of interfacial processes. The first model represents the condition where the adsorbent material has a few active sites; thus, external diffusion or internal diffusion is the rate-controlling step, initial adsorbate concentration is high, and the adsorption process happens in the initial stage [57]. The PSO model represents the conditions when initial adsorbate concentration is low, the adsorbent has many active sites, and adsorption is at the final stage [57]. The intraparticle diffusion model describes adsorption as a process divided into stages, in which adsorbate is first transported from the solution to the outer surface of the polymer and then to the inner pores of the plastic [58]. Accordingly, the PFO and PSO kinetic models are used to evaluate the entire adsorption process, and the IPD is used to show the limiting steps of the adsorption [54]. In all our batch experiments, we used conditions imitating seawater medium for pH, salinity, electrolytes, and temperature. Secondary and spherical MPs presented differences in terms of adsorption capacity, though due to a high difference in size only spherical PP can be directly compared to secondary plastics.

**Figure 7:**
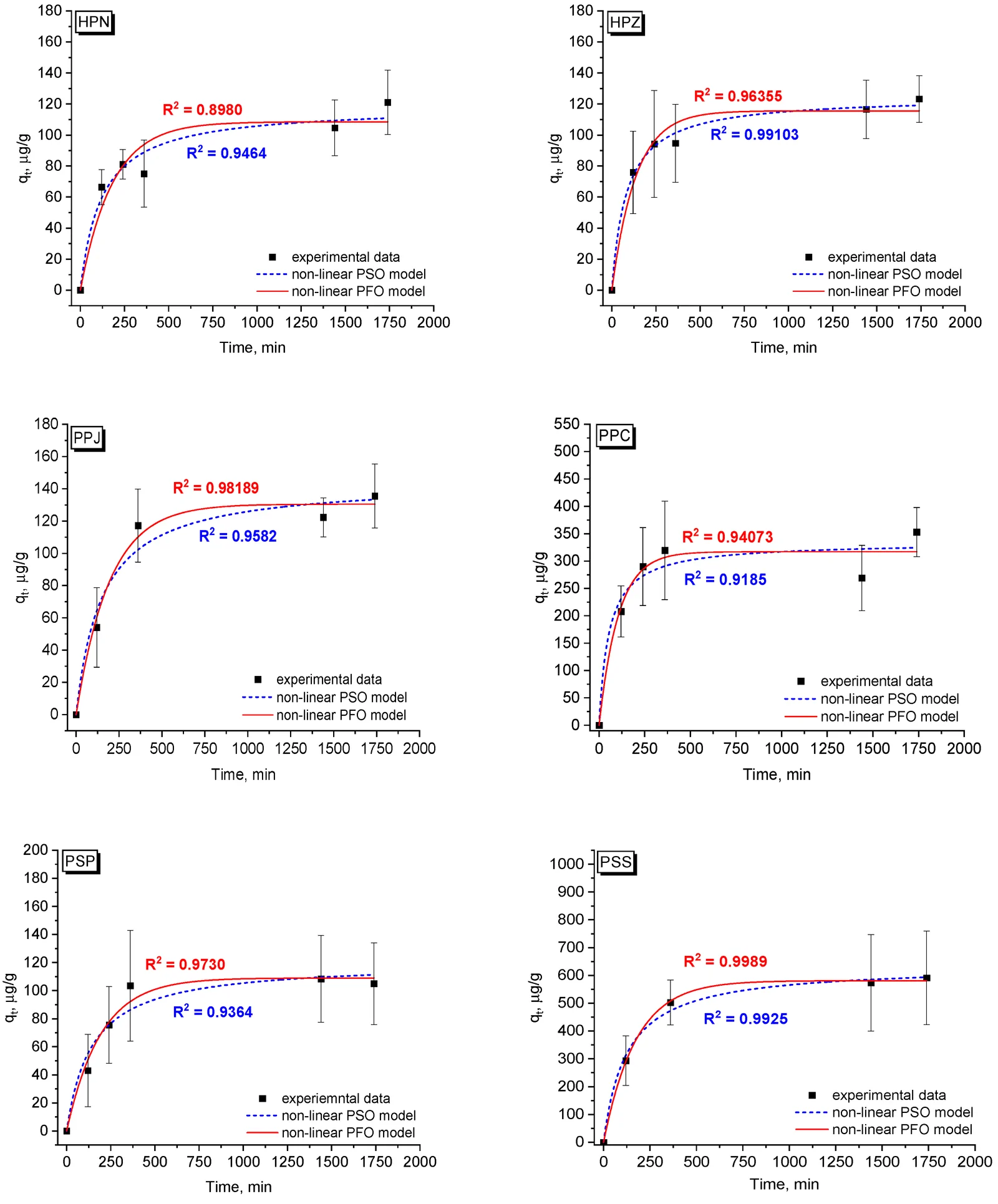
Non-linear fit for PSO and PFO kinetic models for the experimental data obtained during adsorption of Cu(II) on post-consumer at 7‰ (for mesoplastics, spherical MPs and higher salinity see Supplementary Material, Figures S3 and S4). Values are mean± standard error (SE).

As implied by the data in Figure 7 and Table S2 (Supplementary Materials) adsorption kinetics can be relatively well described by both the PFO and PSO models, yielding comparable fitting parameters. The comparable goodness-of-fit suggests that the experimental data do not allow unequivocal discrimination between the two kinetic models. Therefore, adsorption kinetics may involve multiple simultaneous processes, including both diffusion-controlled and surface interaction mechanisms. As far as PSO and PFO models are concerned, it is important to underline that the equilibrium adsorption capacities calculated from these two models were in good agreement with the values determined experimentally. This further suggests that both models adequately describe the adsorption kinetics; however, the kinetic data alone are insufficient to unequivocally identify the dominant adsorption mechanism. We observed poorer fit of experimental data to the PSO and PFO models for secondary MPs at higher salinity, except HPZ and HPN (Supplementary Materials Table S2). This may suggest that the liquid medium itself interfered with the kinetic process.

The adsorption rate and equilibrium time may vary between different types of MPs. Here, for spherical PP and PS, adsorption capacity quickly began to flatten, and after around 200–250 min, the adsorption was not increasing significantly (Supplementary Materials Figure S3). For post-consumer MPs, longer times to reach equilibrium were necessary, between 400 and 500 min (Figure 7). In other studies involving Cu adsorption, equilibrium was reached after 180–300 min for PS and PET microbeads [7,39]. In general, smaller particles can reach equilibrium faster, due to the enhanced diffusion characteristics [40]. In the case of aged MPs and differences in the study conditions, equilibrium may be reached later; for example, in the study by Huang et al. [43], equilibrium was established gradually over 480 min. Comparison of the PFO and PSO rate constants (Supplementary Materials Table S2) showed that the adsorption process was the fastest for PP microspheres, even though their adsorption capacity was the lowest.

**Figure 8:**
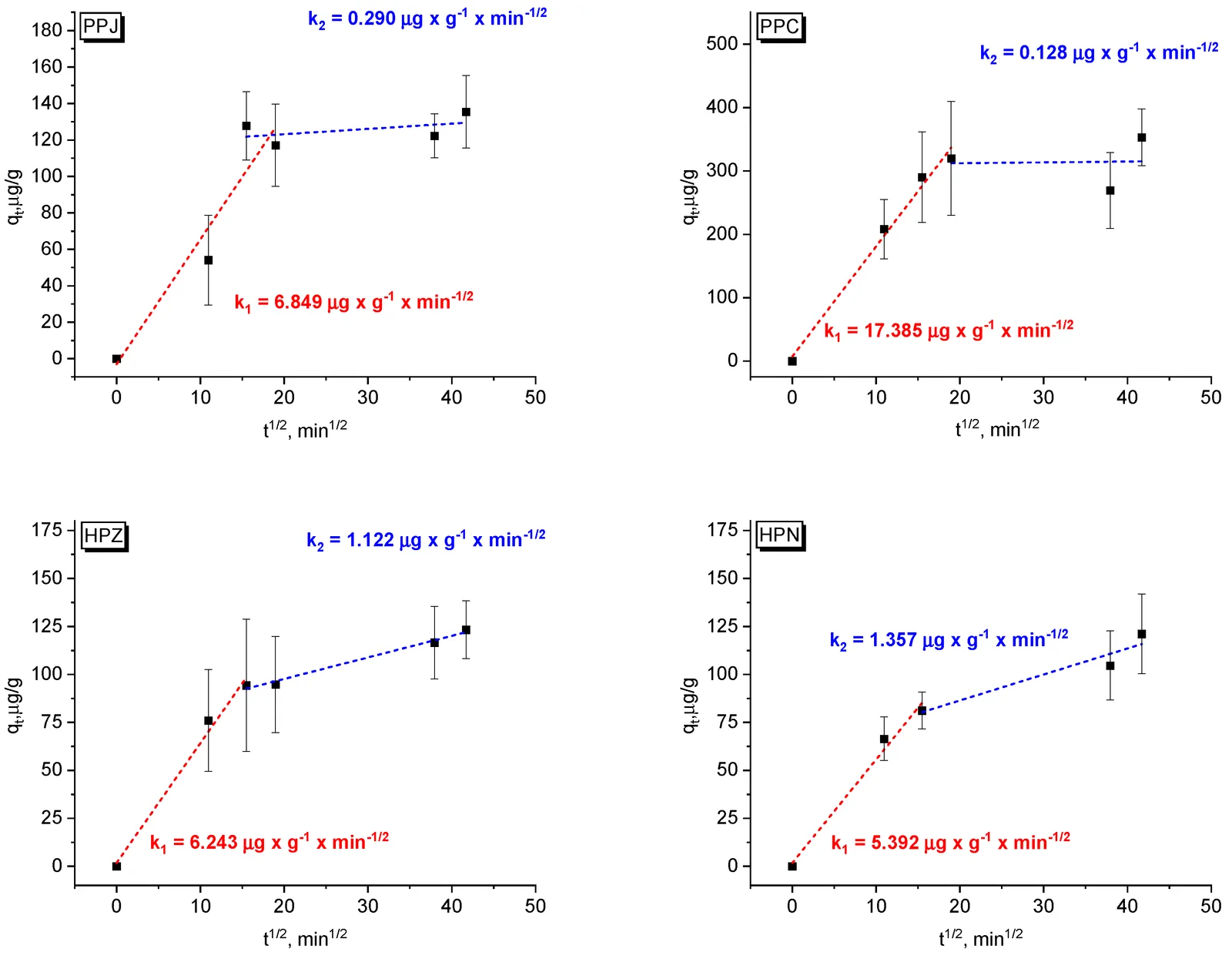

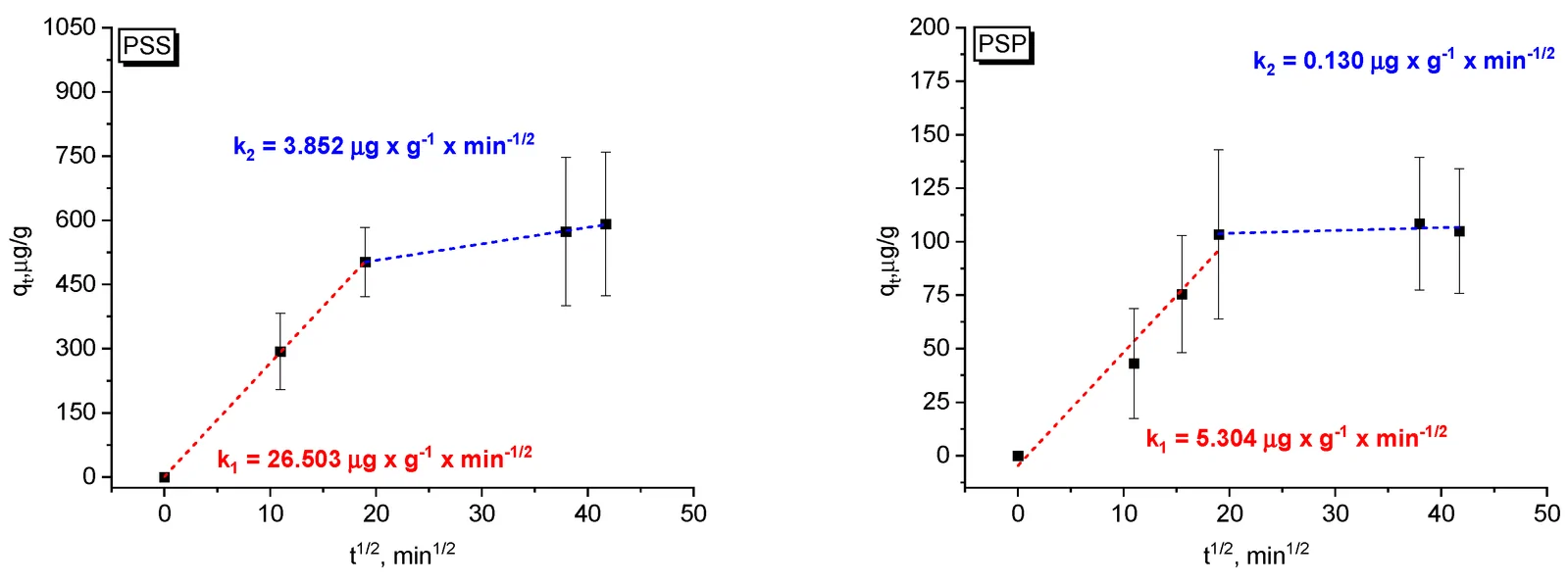
IPD models for microplastics (for salinity 20‰, spherical MPs and mesoplastics see Supplementary Figure S5).

The PFO and PSO kinetic models do not identify the adsorption diffusion mechanism; thus, the intraparticle diffusion (IPD) model was tested to define the rate-controlling steps (Figure 8). The IPD model has been widely used as an adsorption model for variable metal ions (see, e.g., [19,59]). Multilinearity is an indication that more than one mechanism controls the process, and adsorption slows as surface coverage approaches saturation [56]. The first linearity (*k*_1_) refers to external surface adsorption, related to surface- or interface-related processes including complexation and electrostatic interaction [59]. It includes instantaneous adsorption and corresponds to the majority of total adsorbed Cu(II). Since adsorption proceeded quickly at this stage, a high amount of ions was removed from the solution. The second part (*k*_2_) includes the slower part of adsorption, representing the intraparticle diffusion process. It is dominated by the diffusion and adsorption of Cu(II) ions into pores [59]. Slow adsorption suggests saturation of active centers on the plastic surface and a longer diffusion time for Cu ions into the deeper layers of the material structure.

The initial adsorption rate constants (*k*_1_) decrease in the following order: PSS > PPC >> PPJ ≈ HPZ > HPN ≈ PSP, whereas the intraparticle diffusion rate constants (*k*_2_) follow the order: PSS > HPN > HPZ >> PPJ > PSP ≈ PPC. These results indicate that PSS possesses the most favorable adsorption kinetics, exhibiting both the fastest initial adsorption and the most efficient intraparticle diffusion. In contrast, PPC, PPJ and PSP are characterized by fast initial adsorption, but relatively limited intraparticle diffusion. The low value of *k*_2_ indicates that intraparticle diffusion becomes considerably slower after the readily accessible adsorption sites have been occupied. PSP exhibits one of the slowest adsorption processes. Both diffusion constants are relatively low, and the second stage quickly approaches a plateau, indicating limited diffusion within the internal pore structure. HPN and HPZ exhibit slower initial adsorption accompanied by more efficient diffusion within the pore structure. This behavior suggests a slower initial uptake followed by a relatively efficient diffusion of adsorbate molecules into the internal pore structure, possibly reflecting a more accessible porous network.

Increasing the salinity affected the adsorption kinetics differently depending on the adsorbent. The greatest changes were observed in the second diffusion stage, indicating that the ionic strength primarily influenced the transport of adsorbate molecules within the porous structure rather than the initial surface adsorption.

The PSS sample maintained the highest adsorption kinetics under both conditions. The initial adsorption constant increased slightly from 26.5 to 27.3 μg g^−1^ min^−1/2^, while *k*_2_ increased from 3.85 to 4.90 μg g^−1^ min^−1/2^. PSS remained the least affected by increasing salinity and preserved efficient mass transfer throughout both adsorption stages. For PPC, *k*_1_ also increased slightly (17.39 to 18.12 μg g^−1^ min^−1/2^), while *k*_2_ increased considerably from 0.13 to 2.95 μg g^−1^ min^−1/2^. This suggests that elevated salinity facilitated diffusion within the porous structure, despite the lower equilibrium adsorption capacity compared with the low-salinity system.

PSP showed a substantial decrease in the initial adsorption rate (*k*_1_: 5.30 to 2.45 μg g^−1^ min^−1/2^), whereas the intraparticle diffusion constant increased from 0.13 to 0.43 μg g^−1^ min^−1/2^. This behavior suggests that the initial adsorption became slower, while diffusion within the adsorbent particles became relatively more significant under high-salinity conditions. Similarly, for PPJ, the initial diffusion constant decreased from 6.85 to 1.63 μg g^−1^ min^−1/2^, whereas the second-stage diffusion constant changed only slightly (0.29 to 0.31 μg g^−1^ min^−1/2^). The overall adsorption capacity decreased, suggesting that the increased ionic strength possibly reduced the number of available adsorption sites due to competition between dissolved ions and the adsorbate.

In contrast, HPZ exhibited a pronounced decrease in both diffusion constants. The initial adsorption rate decreased from 6.24 to 2.07 μg g^−1^ min^−1/2^, while the intraparticle diffusion constant declined from 1.12 to 0.10 μg g^−1^ min^−1/2^. For HPN, the opposite trend was observed during the initial adsorption stage. The *k*_1_ value increased from 5.39 to 11.25 μg g^−1^ min^−1/2^, whereas *k*_2_ decreased from 1.36 to 0.65 μg g^−1^ min^−1/2^.

Overall, the results demonstrate that increasing ionic strength did not alter the fundamental adsorption mechanism but significantly modified the relative contributions of the individual diffusion stages. Depending on the adsorbent, higher salinity either enhanced or suppressed external mass transfer and intraparticle diffusion, indicating that the effect of dissolved ions strongly depends on the structural and surface properties of the adsorbent.

#### Isotherm Models

Though the physical meaning and the classification of isotherm models are not thoroughly understood, they are often used to model the equilibrium adsorption data to investigate the adsorption mechanisms, the maximum adsorption capacity, and the properties of adsorbents [60]. Many theoretical models exist describing specific physical meaning, including chemical (isotherms describe the monolayer adsorption process), physical (isotherms represent the multilayer adsorption), and ion exchange (model the ion exchange adsorption process) [60]. In previous studies fitting isotherm models, the Freundlich model, which may describe both chemical and physical adsorption [60], was often the better fit when the reaction environment was deionized water or seawater [7,16,39,40] (Supplementary Materials Table S1). For freshwater, the Langmuir model, which is a theoretical monolayer chemical adsorption model [60], could be a better fit [41,53] (Supplementary Materials Table S1). In the case of our samples, under the tested conditions with artificial seawater, we were unable to identify a single matching model that could fit with a high correlation coefficient (all R^2^ < 0.85). The solubility of dissolved Cu in both artificial and natural seawater depends on initial Cu concentration and is triggered by the presence of nucleation sites [47]. We could observe precipitates forming in part of the samples; thus, we suspect that Cu precipitation was responsible for a lack of fit to the chosen models. For this reason, we repeated the experiments using deionized water to eliminate this effect. Here, a good fit for the Langmuir model was observed for PSS, PSP, and HPN (Supplementary Materials Figure S6). The Freundlich model showed a better fit only for the PPC data, although overall the fit was not high. PPJ did not fit well with any of the models tested. The corresponding fitting parameters were recorded in Table 2. The theoretical maximum adsorption capacity (q_m_) in the Langmuir model was highest for PSS (571.43 µg/g) and lowest for HPN (76.39 µg/g). Although we cannot identify any single factor with absolute certainty, the poor fit of the classical adsorption model to polypropylene may be due to the heterogeneous nature of its surface properties, including its high hydrophobicity. It should be noted that a changed medium (deionized water instead of artificial seawater) could affect the adsorption due to a lack of competing ions.

### 2.4. Adsorption in Natural Medium (Baltic Seawater)

Natural water is a complex matrix, containing monovalent and divalent ions, colloids, bacteria, organic matter, macromolecules and other contaminants, with possible high variability between different sites. Multiple factors may cause differences in adsorption, including the presence of metals and organic chemicals, and due to logistical reasons, the number of factors that can be checked is limited. Therefore, we used artificial seawater that mimics the chemical composition of a low-salinity environment, and for comparison, we used natural water collected from three locations on the Baltic Sea to observe changes in the behavior of microplastics in these two environments.

With an initial Cu(II) concentration of 3.3 mg/L added, higher adsorption happened in the presence of natural water when compared to artificial water for PP microspheres (10.5 ± 0.3 µg/g and 4.76 ± 0.86 µg/g, respectively) and for PS (495 ± 19.9 µg/g and 449 µg/g, respectively). Presence of other metals in the natural medium may inhibit adsorption, due to competitive adsorption [7,61], or enhance the adsorption of metal ions, as observed for polyamide MPs [41] and PP [15]. The Baltic Sea is affected by pollution derived from multiple sources, including discharges from ships and recreational boats [62,63]. The samples collected at the harbor and estuary did not show detectable levels of Cu, but lead and chromium were detected in all samples (Supplementary Materials, Table S3).

One batch of samples was left for an additional 2.5 months in Baltic water to evaluate possible changes in concentration after extended exposure, and the potential creation of new functional groups. In these samples, Cu started to slowly desorb, and concentrations dropped from 10.5 ± 0.3 after 24 h to 8.37 ± 1.65 µg/g after 2.5 months for PP; from 495 ± 19.9 to 344 ± 16.8 µg/g for PS and from 58.1 ± 14.5 to 51.4 ± 2.4 µg/g for PE. To verify potential differences in functional groups occurring before and after the adsorption of Cu on PP and PE, we compared spectra before and after the adsorption experiment for samples left for 2.5 months (Supplementary Materials, Figure S7).

The shape of the FTIR spectrum before and after adsorption did not change, and no characteristic additional peaks were observed after Cu (II) adsorption in Baltic seawater. Compared with other studies, new functional groups have been created after a 10-week weathering process under conditions that, besides mechanical weathering with sand, included solar irradiation [17]. Also, in the study by Da Costa et al. [64], after spending 8 weeks in artificial seawater, the PE pellets showed changes in their structure and morphology, especially in the presence of new oxidized groups. Micro-cracks appeared on the surface of the pellets, suggesting accelerated degradation during this time due to the presence of inorganic ions, which can act as oxidation catalysts [64]. Here, although no new functional groups were present, we could observe the first signs of weathering, such as flakes on the surface of PS and rough surface textures on PP and PE (Supplementary Materials, Figure S8), which could further lead to fragmentation.

It is worth noting that in our experiments we used filtered water, with particulate matter removed (except for colloids) and a reduced microbial population. In unfiltered water, these two factors can additionally cause biofilm formation on the surface, which can further affect the adsorption capacity of the material.

### 2.5. Ecological Implications and Study Limitations

Plastic recovered from the environment may contain high levels of trace metals, including Cu (e.g., 645–9094 µg/g on PE pellets [65]). In the case of recovered samples, metals may originate not only from the environment, but may also have been incorporated into their structure during the plastics’ manufacturing process as metal additives intended to improve their properties [15]. However, the values typically observed in the samples are lower, and the maximum adsorption capacities of the samples analyzed in this study were comparable to or higher than the values reported in the literature for plastics found in aquatic environments (e.g., [66,67,68]).

Adsorption to secondary MPs created from everyday life items is different than adsorption to spherical microbeads or pellets. In the marine environment, all MPs are prone to undergoing changes in their surface structure and oxygen-containing functional groups due to the presence of UV radiation and other environmental factors [54]. Secondary MPs, however, additionally contain additives—including antioxidants, dyes, and fillers—that may affect their adsorption capacity. They also vary in terms of versatility and durability and may show signs of aging just from their use, without prior contact with marine seawater. While we did not perform additional aging for our products, signs of oxidation were already observed through X-ray photoemission spectroscopy spectra. The majority of MPs used here had a particle build-up, which suggests that they can release plastic particles, even without additional aging processes.

The choice of medium for the experiment is of particular importance in absorption studies. Artificial seawater can be considered as a representative model of water chemistry of various marine environments. However, precipitation and various forms of copper in water can have a significant impact on the results for different materials and concentrations of compounds. Using more complex aqueous media such as wastewater or irrigation water may also result in metal precipitation [15].

Furthermore, although not covered in this study, bacterial biofilm should be given further attention, as it additionally affects adsorption in natural water. We used homogeneous materials, created from a single type of polymer. Further studies should also use microplastics made from recycled plastic or copolymers, as many consumer products use blends of two or more polymers and those can be found in the marine environment (e.g., [66]). Natural water matrices can increase the adsorption of certain metal ions, but this effect is not consistent across all types of polymers [41]. In natural waters, MPs may additionally be prone to heteroaggregation, which occurs when MPs attach to other solid particles such as natural minerals, colloids and algae [50]. The presence of Cu in water may also enhance adsorption of other compounds, e.g., tetracycline onto PVC microplastics [69]. However, this topic still requires more studies.

Although we have only analyzed Cu adsorption, and other contaminants in water require further study, our analysis has shown that EPS foam was more than four times more susceptible to copper adsorption than hard fragments. Its structure provides more active sites and air pockets, which can be easily filled by water, thereby facilitating adsorption. Flexible PP film pieces had 2.5 times higher adsorption capacity than PP hard fragments. In recent years, more and more research has used MPs created from everyday objects (e.g., [15,34]), but their use for metal adsorption is still not as common as the use of spherical MPs. Using irregular pieces prepared from post-consumer products of sizes found in the environment is necessary to get us a better insight into metal adsorption behavior in the marine environment.

## 3. Materials and Methods

### 3.1. Materials Used

For our experiments, we used two forms of plastic (spherical commercial and irregular post-consumer) divided additionally by size and polymer type. All experiments were performed at room temperature (around 18 °C). We used commercially available white spherical polypropylene (PP, size 2.45 ± 0.05 mm; density ~0.9 g/cc; spherical particles: >95%), polystyrene microspheres (PS, size 9.5–11.5 µm; density: ~1.07 g/cc; spherical particles: >95%), and polyethylene microspheres (PE, size 10–90 µm; density 0.96 g/cc; spherical particles: >90%); all commercial MPs was bought from Cospheric, (Moorpark, CA, USA). Commercial particles had no surface functionalization or coatings added; PS was crosslinked with divinylbenzene (DVB) crosslinking agent. We also used fragments of the post-consumer plastic items (for PP, green vegetable tray and green bottle cups; for PE, green and blue bottle cups, both were high density polyethylene (HDPE); for PS, white clam-shell food container and white coffee cup lid). They were washed, dried at room temperature, and cut into pieces with irregular shapes, as these can be found in the natural environment. Before adsorption experiments, they were sieved and divided into two fractions (between 5 mm and 10 mm as mesoplastics; smaller than 5 mm as microplastics). Abbreviations used in the text for all MP types and its photo are included in Figure 1. Comparing plastic types’ crystallinity based on previous studies, PE and PP are semi-crystalline, while PS is amorphous [54]. As a result of using different products, we obtained different types of MPs based on their morphological characterization, i.e., hard fragments (made of three types of bottle cups), films (thin flat flexible plastic made of PS cup lids and PP trays), and foam (made of EPS clamshell containers) (divided based on GESAMP report [70]).

Batch experiments were carried out in 40 mL glass vials, thoroughly cleaned with diluted 10% nitric acid (65% Suprapur, Sigma-Aldrich, St. Louis, MO, USA). The experiments were carried out using artificial seawater and fortified real environmental waters. For this purpose, in May 2023, we collected Baltic surface seawater at three sites on Polish coastline affected in a smaller or bigger scale with shipping activity: Górki Zachodnie (54°22′14.5″ N 18°46′38.7″ E; place where Vistula river meets Baltic water), beach at Nowy Port Gdańsk (54°24′12.6″ N 18°41′10.0″ E) and Gdynia Port (54°31′04.5″ N 18°33′02.5″ E). Natural waters were transported to the laboratory and kept under cool conditions. No pH adjustment of the natural waters was conducted to provide environmentally relevant conditions for the experiments (conductivity between 10.08 and 10.9 mS, pH between 7.31 and 7.85). Fresh artificial seawater was prepared based on the composition provided by Wang et al. [71]. The electrolyte mixture was prepared by mixing NaCl (62.26 g; POCH), MgCl_2_·6H_2_O (12.82 g; Sigma-Aldrich), MgSO_4_·7H_2_O (14.39 g; POCH), CaCl_2_·2H_2_O (3.30 g; Sigma-Aldrich), KCl (1.74 g; POCH), and NaHCO_3_ (0.49 g; POCH). Artificial seawater was made by dissolving the salt mixture in deionized water to a concentration of ≈7 g/L (all volume concentrations are reported for 18 °C), which is the average salinity of the surface layers of the Baltic Sea. The synthetic seawater solution was prepared with deionized water obtained from a Hydrolab water purification system. Copper used in the experiments was in the form of soluble metal salt (CuSO_4_ × 5H_2_O, Sigma-Aldrich).

### 3.2. Adsorption Kinetics

#### 3.2.1. Experiments in Artificial Seawater

Batch adsorption experiments were carried out to study the adsorption characteristics of Cu(II) on MPs. For the post-consumer plastic, both the micro- and mesofraction were firstly placed in artificial seawater (pH 7.46–7.91; conductivity 11.53–11.65 mS). For that purpose, they were weighed separately into vials with 30 mL of freshly prepared artificial seawater (7‰). Vials were put on an orbital shaker and delicately mixed (30 rpm) for 7 days to equilibrate. After 7 days, pH was checked (values between 7.48 and 7.96). Then, 0.3 mL of stock solution (CuSO_4_ × 5H_2_O = 2 g/L) was added to the samples, reaching 5 mg/L of Cu(II) and vials were mixed on the orbital shaker at 150 rpm to imitate wave motion in the water. This concentration was chosen to ensure that detection limits are reached even at low adsorption levels. After Cu addition, the pH in all vials dropped to around 7.0. One mL of solution was collected after 0, 2, 4, 6, 24, and 28 h. No metal precipitation was observed during the time of the experiment. We analyzed 4 repetitions of each MP type, as we expected higher variability due to irregular shape. Analysis included blanks with only water and Cu added to check the adsorption into glass vials. After completing the experiment, we mineralized the samples to determine the percentage of Cu adsorbed onto the surface of the plastic and to compare this with the amount calculated from the collected water. For this purpose, the plastic was filtered from the solution, poured with deionized water, and dried. Samples were mineralized at 95 °C for 8 h on a hot plate with 4 mL of HNO_3_ (65% Suprapur, Sigma-Aldrich). After mineralization, samples were diluted to 10 mL with deionized water before analysis by the ICP-MS analytical technique. The results were corrected for the difference in Cu content. Raw post-consumer plastics were additionally mineralized to check possible Cu release from untreated plastic. Concentrations were below the detection limit, which confirmed that they did not release Cu(II) into the solution.

Mean mass for all MP types was: PPJ 18.6 ± 2.7 mg; PPC 12.0 ± 1.2 mg; HPZ 17.1 ± 2.9 mg; HPN 13.1 ± 1.3 mg; PSS 11.1 ± 1.2 mg; PSP 16.2 ± 1.2 mg; and for mesofraction—PPC 50.4 ± 10.6 mg; HPZ 81.0 ± 10.6 mg; PSS 42.8 ± 0.5 mg.

For comparison purposes, we prepared a second batch which included spherical microspheres of three polymers (mean mass of PP 76.3 ± 0.7 mg; PS 18.2 ± 2.4 mg; PE 28.0 ± 4.8 mg). Each type was prepared in triplicate using the same method as for post-consumer plastic. One mL of solution was collected within 15 s after Cu addition (0) and after 2, 4, 24, and 29 h. A set of six blanks was also included.

Additionally, to study the influence of salinity on the sorption behavior, we prepared water of initial pH = 8 and salinity 20‰. The salinity corresponded to the levels found in the surface waters of the Baltic Sea (7‰) and to the higher salinity found, for example, in the Danish straits or estuaries, where freshwater from rivers mixes with seawater (20‰). This batch included post-consumer MPs and spherical MPs, which were analyzed using the above methodology. After 4 h of the experiment, we could observe some metal precipitation in the vials. Usually, the metal concentration on the MPs’ surface is calculated only based on the collected water sample; however, here we mineralized all the samples, and the concentration of the Cu actually adsorbed onto the surface of the MPs was corrected.

Samples for isotherms were prepared in duplicates for all secondary MPs in artificial seawater. However, due to precipitation in some samples and a poor fit to chosen isotherm models, experiments were repeated using deionized water. Six data points were used in the study, with the Cu concentration in the samples ranging from 0.5 to 5 mg/L. A separate set of blank samples was prepared for each concentration; the value obtained from these served as the initial concentration. After 24 h, 1 mL of sample was collected for analysis.

#### 3.2.2. Experiments in Baltic Seawater

Natural water was filtered with a 0.45 µm filter (Whatman, Maidstone, UK) to remove potential MP particles and suspended matter and checked for initial metal levels, conductivity, and pH. The goal of using natural matrix was to check the adsorption capacity of MPs after they spend some time in natural seawater and to compare potential interferences with artificial seawater (for spherical MPs only). Concentrations of the selected metals (Pb, Zn, Cu, Al, Cd, Cr, Se) in the Baltic seawater were measured by the ICP-MS analytical technique, representing the background metal concentration (Supplementary Materials, Table S3). MPs were weighed and placed in vials, then 30 mL of water was added. We included blanks with only water added to vials without MPs. Vials were placed on an orbital shaker at 150 rpm. To detect any changes in water conditions, samples were collected after 1.5 h, 24 h, 8 days, and 14 days. After that time, 200 µL of Cu stock solution was added, and to compare with artificial seawater, additional vials with the same concentration used were prepared (following the above methodology). Vials were shaken at 150 rpm, and the last sample was collected after 24 h. pH was checked before and after Cu addition on blank samples. One batch was left for another 2.5 months on a shaker to check adsorption and to compare changes in the structure after 14 days and 2.5 months in water (78 days in total). The presence of coexisting ions was systematically investigated.

#### 3.2.3. MPs Characteristics and Analytical Method

To better analyze the mechanism of adsorption behavior, the microscopic morphology and structure of MP particles before and after adsorption were investigated by Scanning Electron Microscope (model FEI Quanta FEG 250; Thermo Fisher Scientific, Hillsboro, OR, USA). Measurements were carried out in high vacuum mode using a secondary electron detector (ETD), with an accelerating voltage of 10 kV, and samples were coated with a 10 nm gold layer before measurement. To confirm post-commercial plastic types and identify the surface structure and functional groups, we used Fourier transform infrared spectroscopy equipped with attenuated total reflection equipment (FTIR-ATR Thermo Scientific Nicolet iS10; Waltham, MA, USA). FTIR spectra were acquired within a wavenumber range from 4000 to 400 cm^−1^.

The detailed chemical composition of the sample surface was analyzed by X-ray photoemission spectroscopy (XPS) (Scienta Omicron, Uppsala, Sweden). Samples were measured at room temperature, under ultra-high vacuum conditions and pressures below 10^−5^ Pa. The photoelectrons were excited by a Mg-Kα X-ray source operated at 15 kV and 300 W. An Argus hemispherical spectrophotometer equipped with a 128-channel detector was used for electron energy measurements. Recorded spectra were calibrated to obtain the C-C peak at 285 eV. The data analysis was performed with the CASA XPS software (version 2.3.15), using Shirley background subtraction and the Gauss–Lorentz curve fitting algorithm by the least-squares method—GL (30). Data analysis was supported using Tompkins and Fisher [72].

The surface hydrophobicity of the post-consumer microplastics was measured using the static water contact angle method on a Drop Shape Analysis System DSA 10 Mk2, Krüss (Hamburg, Germany). The instrument was equipped with an automated microliter syringe and a digital camera. Briefly, 5 μL of deionized water was dispensed on each sample surface, and measurement was carried out within 5 s. The measurement was carried out at 25 °C and was repeated at least six times for each sample at different locations on the plastic surface. For green PP trays and white PS trays, separate analyses of the inner and outer parts of the tray were performed, as differences in their structure were observed.

The Brunauer–Emmett–Teller (BET) specific surface area was determined by nitrogen adsorption using an Autosorb iQ adsorption analyzer (Quantachrome Instruments, now Anton Paar; Boynton Beach, FL, USA), model 7, equipped with ASiQWin 5.2 software. Before analysis, a suitable amount of each microplastic sample was degassed at 40 °C for 24 h to eliminate adsorbed moisture and volatile impurities. After degassing, the sample cell was cooled and immersed in liquid nitrogen, where nitrogen adsorption–desorption isotherms were recorded. The data obtained were processed using ASiQWin 5.2 software. The specific surface area was computed using the linear Brunauer–Emmett–Teller (BET) model, applying the appropriate linear region of the adsorption isotherm.

The metal concentration in aqueous solution and mineralized samples was analyzed by inductively coupled plasma-mass spectrometry (ICP-MS). The analysis of Pb, Zn, Cu, Al, Cd, Cr, and Se was performed using an ICP-MS 2030 (Shimadzu, Kyoto, Japan) equipped with Collision Cell Technology. All instrumental parameters set for the analysis were optimized to achieve the best performance for the analysis of chosen elements. Measurement conditions can be found in Supplementary Materials (Table S4).

#### 3.2.4. Data Analysis

The adsorption capacity of each MP was calculated based on the equation (Equation (1)):

(1) $$q_{t}=\frac{V(C_{0}-C_{t})}{m}$$

where *q_t_* (µg/g) and *C_t_* (µg/L) are the adsorption capacity and concentration of Cu ions at time *t*, respectively; *C*_0_ (µg/L) is the initial concentration of metal ions; *V* (L) is the volume of solution; and *m* (g) is the mass of plastic used.

Adsorption kinetic parameters were determined by using non-linear pseudo-first order (PFO) (Equation (2)) and pseudo-second order (PSO) (Equation (3)) models [73].

(2) $$q_{t}=q_{e}(1-e^{-k_{1}t})$$

(3) $$q_{t}=\frac{k_{2}q_{e}^{2}t}{{1+k}_{2}q_{e}t}$$

where q(t) and $q_{e}$ are the amount of adsorbate adsorbed at any given time, t, and at equilibrium, respectively; and $k_{1}$ and $k_{2}$ correspond to the adsorption rate constant for the pseudo-first order and pseudo-second order model, respectively.

Adsorption kinetics were also analyzed using the Weber–Morris intraparticle diffusion (IPD) model (Equation (4)) [74].

(4) $$q_{t}=K_{id}t^{\frac{1}{2}}+C$$

where $q_{t}$ is the adsorption capacity at any time (µg/g), $K_{id}$ is the intraparticle diffusion rate constant (µg g^−1^ min^−1/2^), t is the time taken for the adsorption process (min) and C is a constant for any experiment (µg/g) that gives an idea about the thickness of the boundary layer. When a graph of $q_{t}$ is plotted against $t^{\frac{1}{2}}$, a linear graph is obtained with regression linear coefficient, $R^{2}$, close to unity. The slope of the graph reveals the intraparticle diffusion rate constant, $K_{id}$, while the intercept of the graph stands for C.

Experimental data were fitted to Langmuir and Freundlich adsorption models. Adsorption isotherm parameters were determined by using the linearized form of the Langmuir (Equation (5)) and Freundlich (Equation (6)) [75].

(5) $$\frac{C_{e}}{q_{e}}=\frac{1}{q_{m}}\left(C_{e}\right)+\frac{1}{q_{m}K_{L}}$$

(6) $$\log_{10}q_{e}=\frac{1}{n}\log_{10}C_{e}+\log_{10}K_{F}$$

In the case of the linear form of Langmuir model (Equation (5)), $q_{e}$ is the amount of adsorbate per unit mass of adsorbent at equilibrium (µg/g), $K_{L}$ is the adsorption capacity constant (L/g), $C_{e}$ is the concentration of adsorbate at equilibrium (µg/L), and $q_{m}$ is the maximum adsorption capacity (µg/g).

In the case of the linear form of Freundlich model (Equation (6)), where $q_{e}$ is the amount of adsorbate per unit mass of adsorbent at equilibrium (µg/g), $K_{F}$ is the adsorption capacity constant (L/g), $C_{e}$ is the concentration of the adsorbate at equilibrium (µg/L), n is the heterogeneity factor, and $\frac{1}{n}$ is the adsorption intensity.

The kinetics of Cu adsorption were evaluated using a linear mixed-effects model implemented in the R package lme4. Adsorption capacity (µg/g) was used as the response variable, with material type and incubation time included as fixed factors together with their interaction. Because measurements at different time points were repeatedly collected from the same vial, vial identity was incorporated as a random intercept. Models were fitted using restricted maximum likelihood (REML). The significance of fixed effects was assessed by Type III ANOVA with Satterthwaite’s approximation of degrees of freedom using the lmerTest package. Post hoc comparisons were performed using estimated marginal means and Tukey-adjusted pairwise contrasts (emmeans package). A separate analysis was carried out to compare the effect of salinity on adsorption kinetics. Results were considered statistically significant at *p* < 0.05. R version 4.5.2 was used for analysis.

## 4. Conclusions

Both secondary MPs and Cu(II) ions coexist in the seawater; thus, understanding their behavior, mechanism of interaction, and effects under environmental conditions is crucial to understanding the ecological risks they can cause. Given the complexity of any aquatic environment and multitude of factors that affect adsorption processes, the experiments were carried out using artificial seawater at environmentally relevant pH and temperature.

Main conclusions based on used materials:

(a) Adsorption kinetics depend to a large extent on the type of material. PP is generally considered a material of low adsorption capabilities, as proved in multiple studies on microbeads of pellets, and on microspheres in this study. However, surface properties, and the form of secondary plastic (hard fragments vs. film) may significantly change its adsorption capability, as here flexible film made from PP had much higher adsorption capacity than other forms. For PS, a significant difference could also be observed depending on the type of PS used. Foamed EPS had much higher potential for water and contaminant adsorption than film made from XPS. Hard fragments made from both PP and HDPE bottle cups showed highly comparable and low levels of adsorbed Cu.
(b) The foamed container made from EPS exhibits the greatest capacity for Cu adsorption and adsorbs significantly more Cu(II) than most other materials. Modification to the meso-form causes a very sharp decline in adsorption for foam EPS, whereas for other materials this difference was not statistically significant.
(c) All secondary MPs had signs of aging and more oxygen-containing groups, resulting in a stronger ability to interact with Cu ions.
(d) For some materials, high variability between vials limits the ability to demonstrate significant differences despite large differences in means.

Secondary MPs are ubiquitous in the marine environment in the form of fragments, films, foams, and other forms not covered by this study. The effect of added additives in post-consumer MPs should be further studied. Both the pseudo-second- and pseudo-first-order equations fitted well to experimental data, suggesting that more than one mechanism is engaged in adsorption processes. Equilibrium was reached in a relatively short contact time for microspheres (around 200 min for PP and PS), while twice as long a time was necessary for most post-consumer MPs. This study highlights the need to use more realistic sample types (homogeneous polymers and co-polymers) as secondary MPs in further studies but also the difficulties connected with the use of irregular non-uniform fragments.

## Figures and Tables

**Table 1:** Comparison of the properties of secondary plastics.

Plastic Type	Water Contact Angle	Type	Color	Buoyancy in Seawater	Mean ± SE (Standard Error) Adsorption Capacity After 28 h [µg/g]
PSS	89.7 ± 5.7° (outer side), 83.2 ± 8.9° (inside)	Foam	white	Float at the surface	Micro: 591.4 ± 168.0 Meso: 94.3 ± 22.2
PSP	91.2 ± 5.2°	Flexible film	white	Sink to the bottom	Micro: 105.0 ± 29.1
HPZ	85.1 ± 2.2°	Hard fragment	green	Float at the surface	Micro: 123.3 ± 15.1 Meso: 79.1 ± 6.6
HPN	87.4 ± 4.9°	Hard fragment	blue	Float at the surface	Micro: 121.1 ± 20.8
PPC	101.2 ± 1.6° (outer side), 90.2 ± 5.9° (inside)	Flexible film	dark green	Float at the surface	Micro: 352.9 ± 45.0 Meso: 229.2 ± 59.8
PPJ	107.8 ± 2.5°	Hard fragment	green	Float at the surface	Micro: 135.5 ± 20.0

**Table 2:** Adsorption parameters derived from linear adsorption isotherm models in deionized water.

Sample	Langmuir Model			Freudlich Model			
	K_L_ (L/µg)	q_m_ (µg/g)	R^2^	K_F_ (µg/g) (L/µg)^1/n^	n	1/n	R^2^
PSS	0.0010	571.43	0.9559	17.84	2.55	0.39	0.8999
PSP	0.0115	143.06	0.9909	n/a	n/a	n/a	0.0004
PPC	n/a	n/a	0.0072	0.11	1.18	0.85	0.7341
HPN	0.0040	76.39	0.9595	n/a	n/a	n/a	0.0027
PPJ	0.0009	81.90	0.5783	n/a	n/a	n/a	0.0920

n/a stands for ‘not applicable’ due to the very poor fit of the experimental data to the given adsorption model.

## Data Availability

The original contributions presented in this study are included in the article/Supplementary Materials. Further inquiries can be directed to the corresponding author.

## Supplementary Materials

The following supporting information can be downloaded at: https://www.mdpi.com/article/10.3390/molecules31162873/s1, References [6,7,15,16,19,39,40,41,42,53,76,77] are cited in the Supplementary Materials.

## Abbreviations

The following abbreviations are used in this manuscript:

PSO	pseudo-second-order model
PFO	pseudo-first-order model
PA6	polyamide 6 (nylon)
IPD	intraparticle diffusion model
SUP	single used product
EPS	expanded polystyrene
XPS	extruded polystyrene
PP	polypropylene
HDPE	high-density polyethylene
PS	polystyrene
PVC	polyvinyl chloride
BET	Brunauer–Emmett–Teller
SEM	Scanning Electron Microscope
FTIR	Fourier transform infrared spectroscopy
ICP-MS	inductively coupled plasma-mass spectrometry
