# Supplementary material for: Adsorption of Copper Ions to Secondary Microplastics in Seawater
Source: Molecules. 2026 Aug 17;31(16):2873. doi: 10.3390/molecules31162873 (PMC13515476; doi:10.3390/molecules31162873)
Supplement: Supplementary file 1 [file molecules-31-02873-s001.zip › molecules-4398968-supplementary.pdf]

Supplementary Material

Adsorption of Copper Ions to Secondary Microplastics in Seawater

Aneta Dorota Pacyna-Kuchta [1,*], Jakub Karczewski [2], Anetta Zioła-Frankowska [3], Lukasz Wolski [3], Marcin Łapiński [2], Kinga Kujawska [2] and Marcin Frankowski [3]

[1] Department of Biotechnology and Microbiology, Faculty of Chemistry, Gdańsk University of Technology, 11/12 Narutowicza Street, 80-233 Gdańsk, Poland
[2] Advanced Materials Centre, Faculty of Applied Physics and Mathematics, Gdańsk University of Technology, 11/12 Narutowicza Street, 80-233 Gdańsk, Poland; jakub.karczewski@pg.edu.pl (J.K.); marcin.lapinski@pg.edu.pl (M.Ł.)
[3] Faculty of Chemistry, Adam Mickiewicz University Poznań, Uniwersytetu Poznańskiego 8 Street, 61-614 Poznań, Poland; anetta.ziola-frankowska@amu.edu.pl (A.Z.-F.); wolski.lukasz@amu.edu.pl (L.W.); marcin.frankowski@amu.edu.pl (M.F.)
* Correspondence: aneta.kuchta@pg.edu.pl

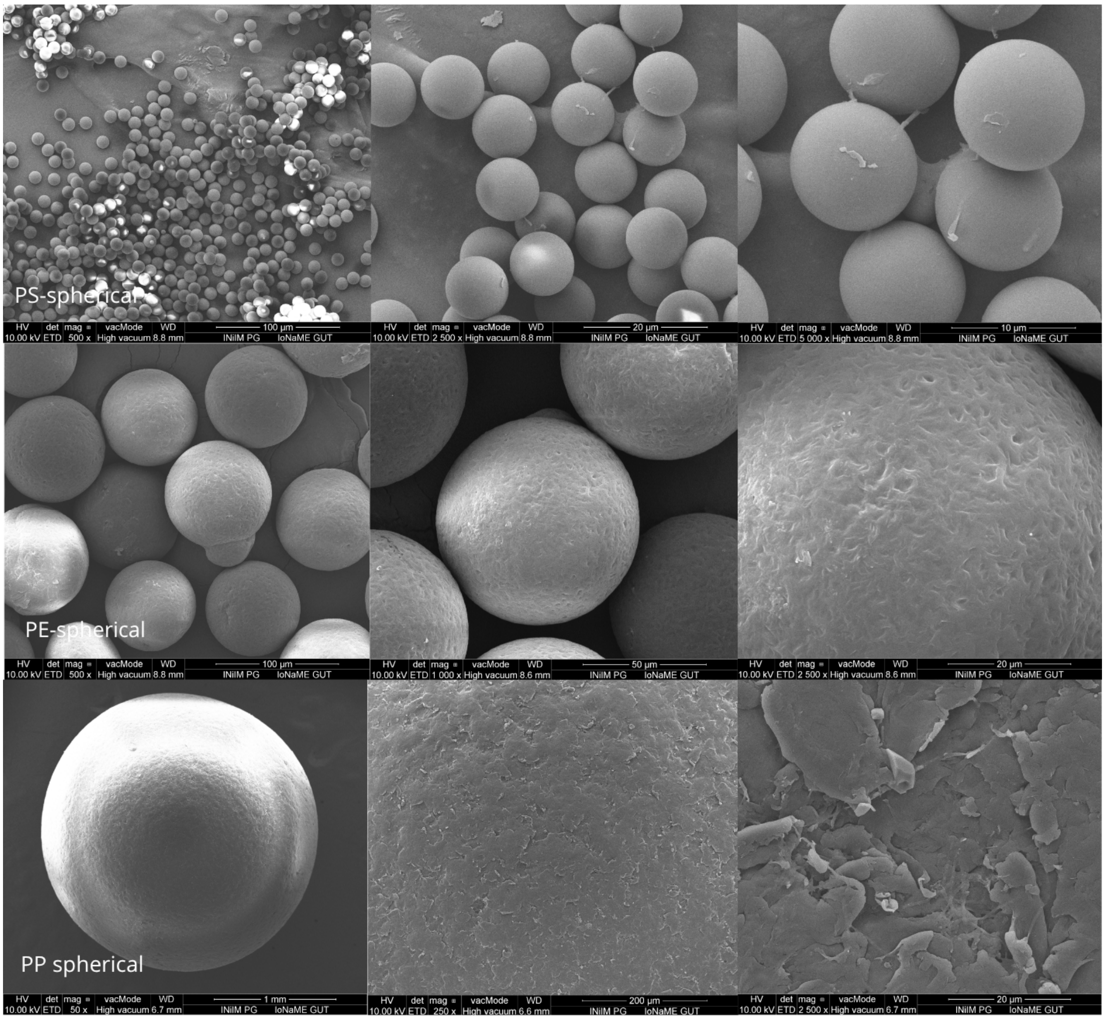

Figure S1. SEM morphology on spherical MPs after adsorption experiment; PS (500x, 2500x and 5000x), PE (500x, 1000x and 2500x), PP (50x, 250x and 2500x).

Text S1.

Commentary to BET analysis

The nitrogen adsorption isotherms obtained for the secondary microplastics and spherical PP showed non-physical behaviour in the low relative pressure range, including a very low or negative apparent amount of adsorbed gas, which made it impossible to reliably determine the specific surface area using the BET method. In order to verify whether the observed effect could result from improper sample preparation, a blank test was performed, i.e. an adsorption isotherm for an empty measuring cell under identical experimental conditions. The blank test measurement showed the presence of a significant, systematic adsorption signal. It should be emphasised that, with a correctly determined free volume and sufficient sensitivity of the apparatus, the isotherm for an empty cell should take values close to zero across the entire range of relative pressures. The observed non-zero signal for the 'empty' measurement indicates that the results obtained are not related to the preparation of the sample or its physicochemical properties, but result from the limitations of the measurement method. Consequently, it was concluded that the specific surface area of the tested samples was below the detection limit of the nitrogen adsorption method at 77 K. For materials with low specific surface area, the literature recommends the use of krypton adsorption, but this was not possible in the present study due to the lack of access to krypton and time constraints.

Table S1. Examples on previously done experiments on Cu(II) adsorption onto MPs.

**Cu concentration/ conditions for main adsorption studies**	**Type of MPs used**	**Adsorption environment**	**Factors that impacted adsorption**	**Best fit kinetic model**	**Best fit isotherm model**	**Proposed mechanism**	**Reference in the main text**
Cu leached from plates painted with antifouling paint	PS beads(0.7-0.9 mm), PVC fragments (1.6 * 0.8 mm)	Seawater	Type of MPs (PVC>PS)	first-order approach to equilibrium model	-	direct adsorption of cations or complexes onto charged sites or neutral regions of the MPs surface	[6]
Cu added to artificial seawater (0.5 mg/L of metal solution)	PS and PET pellets (25 µm and 180 µm)	Seawater (high salinity)	the dosage and particle size of particles, pH, presence of Pb ions	pseudo-second-order model	Freundlich model	physical adsorption	[7]
Cu added to deionized water (0.5-32 ppm)	PE, PET, PP, PVC and PS (<5mm fragments)	Milli-Q water, seawater, urban wastewater, irrigation water	physical-chemical properties of MPs	-	Langmuir model	chemical adsorption	[15]
Cu added to distilled water (0.10-50.00 mg/L)	chlorinated polyethylene (CPE), PVC, LPE, HPE	Distilled water	pH, ionic strength	-	Freundlich model	Electrostatic interaction and surface complexation	[16]
Cu added to water (0.25-5 mg/L)	PE (60–150 µm)	Water, sewage outlet	pH, competition interactions	Intra-particle diffusion model; film diffusion model	Freundlich model	intra-particle diffusion (virgin MP) and film diffusion (MP with biofilm); complexation interactions	[19]
Cu added to filtered seawater (0.01- 2 mg/L)	PS, PE (15 to 30 µm)	Seawater	surface chemical functional groups, aging	pseudo-second-order model	Freundlich model	chemisorption	[39]

Cu added to DI and artificial seawater (10 mg/L)	PS (130-520 nm), raw and UV-aged	Deionized and artificial seawater	Salinity, UV-aging, temperature, particle size	Pseudo-second order model	Freundlich model	electrostatic and coordination bonding	[40]
Cu added to natural water (3 mg/L)	Polyamide (PA)	Freshwater	pH, salinity, and the concentration of citric acid and humic acid	Elovich and pseudo second-order	Langmuir model	surface complexation, chemisorption	[41]
Cu added to water (5 mg/L)	PE, PP, and PET pellets crushed to smaller size (2–5, 0.9–2, and <0.9 mm)	Deionized water	Particle size	Pseudo-second order model	-	-	[42]
Cu added to artificial freshwater (50 μg/L of metal solution)	PP, PS, and PVC particles (spheres maximum sizes of 100 μm)	Freshwater	temperature and salinity	pseudo-second-order; intra-particle diffusion	Langmuir model	monolayer physical adsorption	[53]
Cu added to water (1 mg/L)	PP, PE virgin pellets	Deionized water	Exposure time, pH, salinity	-	-	-	[76]
Cu added to filtered water (5 μg/L)	PE pellets new and aged	Freshwater, seawater	aging	-	non-linear Langmuir (seawater) or Freundlich (river)	Cu ions and additional organic complexes interaction with pellet surface	[77]

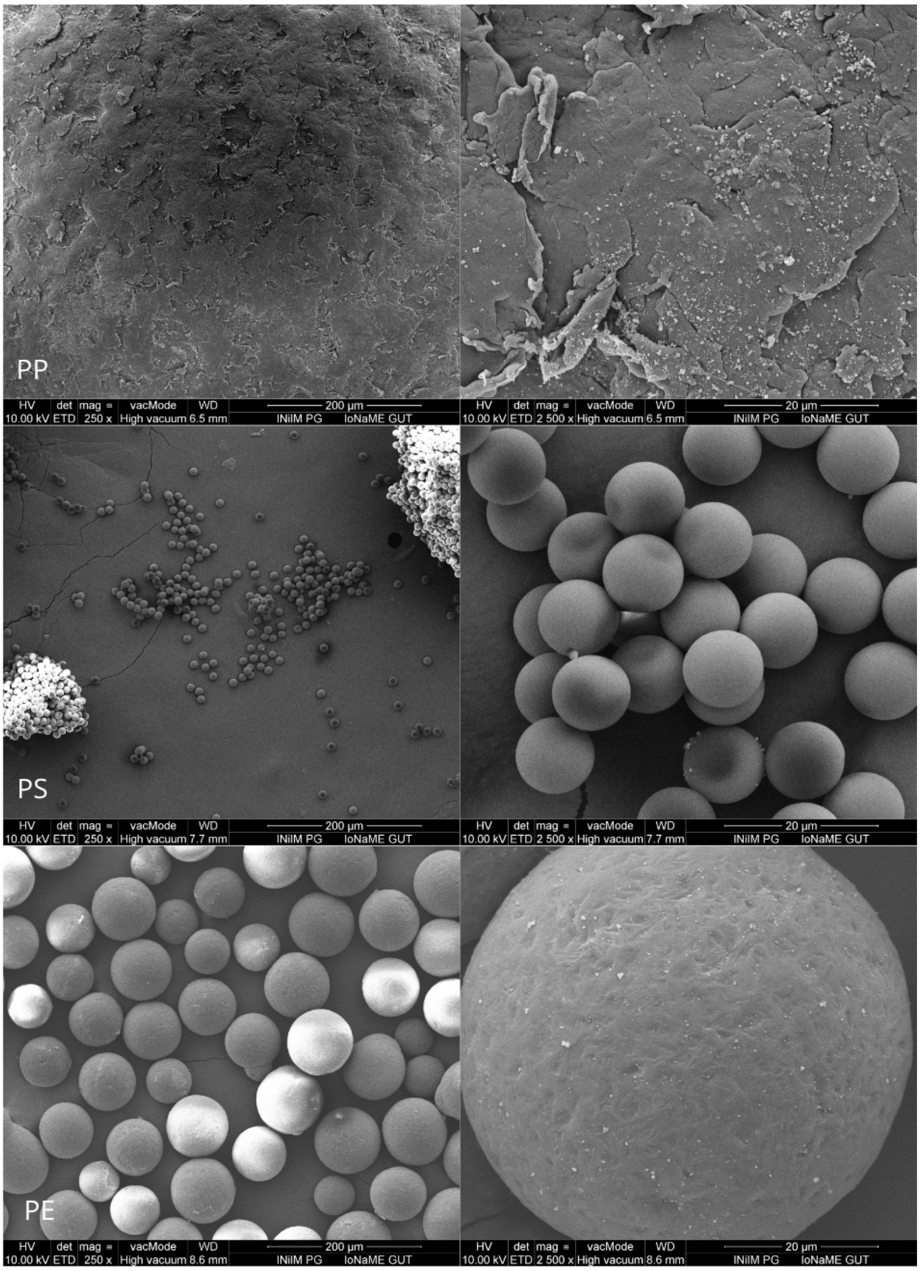

Figure S2. SEM after adsorption study in seawater (20 ‰), magnification 250x and 2500x.

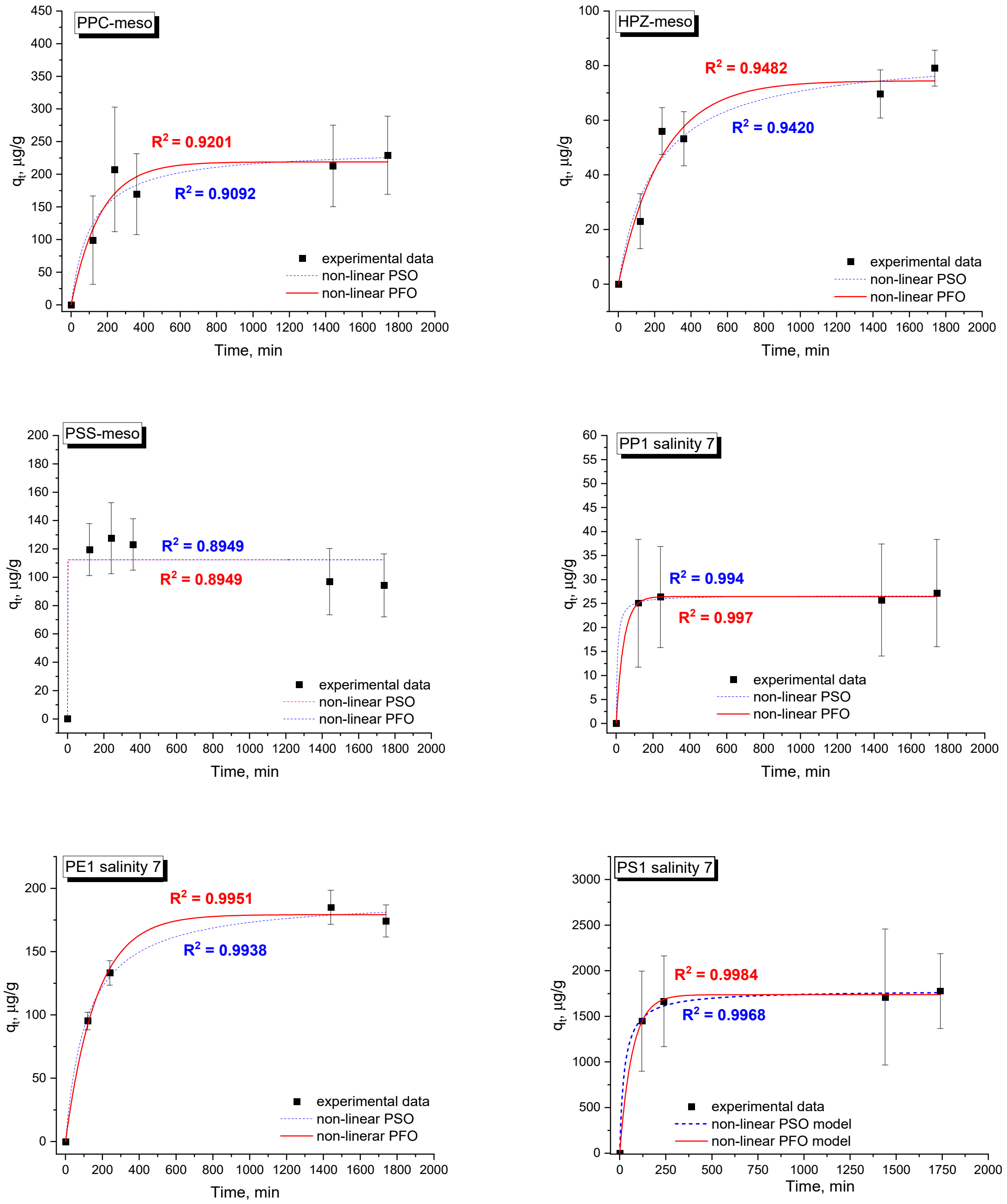

Figure S3. Non-linear fit for PSO and PFO kinetic models for the experimental data obtained during adsorption of Cu(II) on post-consumer mesoplastics and spherical MPs (7‰).

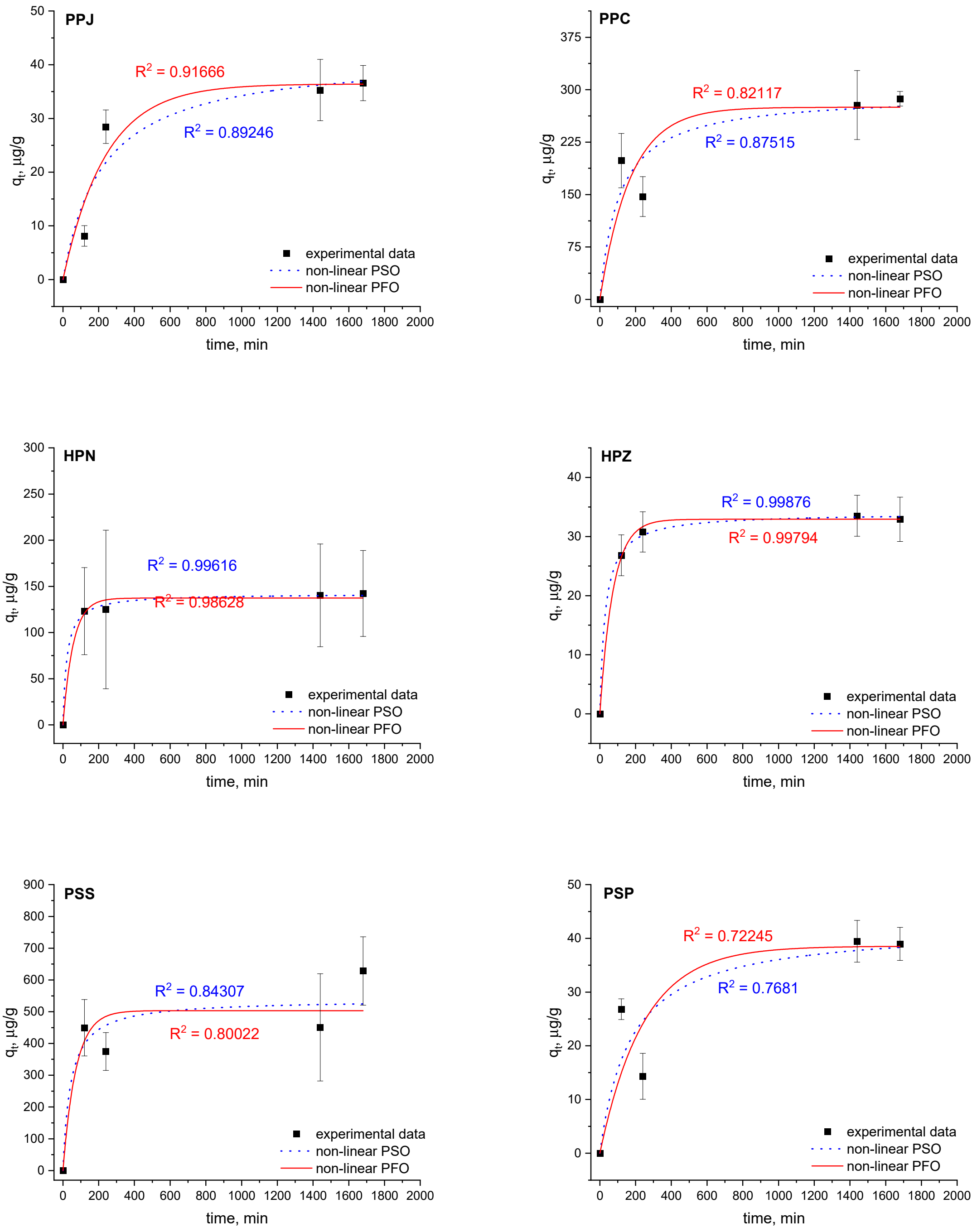

Figure S4. Continuation on the next page.

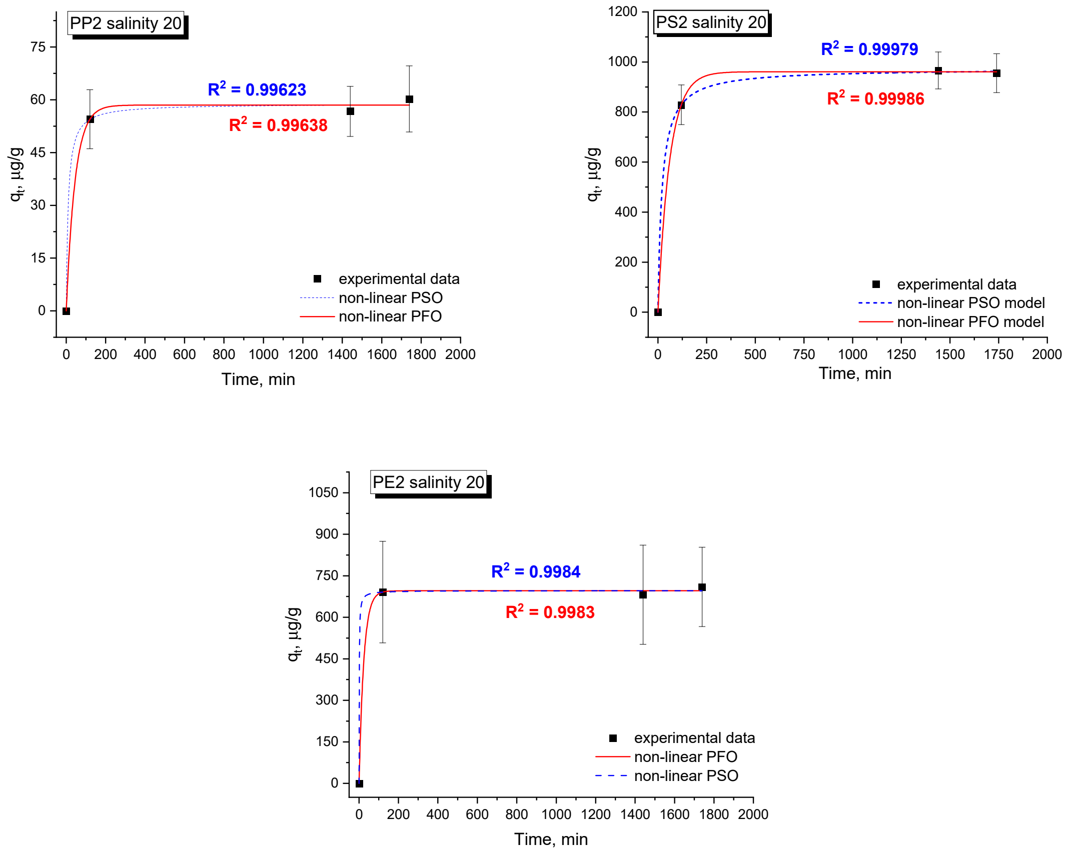

Figure S4. Non-linear fit for PSO and PFO kinetic models for the experimental data obtained during adsorption of Cu(II) on post-consumer microplastics and spherical MPs for higher salinity (20‰).

Table S2. Parameters for non-linear PFO and PSO models

Sample	q_e (exp) [μg/g]	PFO model			PSO model		
		k [min^{-1}]	q_e (cal) [μg/g]	R^2	k_2 [$g \cdot \mu g^{-1} \cdot min^{-1}$]	q_e [μg/g]	R^2
PPJ	135.47	0.00515	130.49	0.98189	0.00005	144.84	0.95821
PPC	319.58	0.00948	317.11	0.94073	0.00006	334.33	0.91853
HPZ	123.28	0.00740	115.58	0.96355	0.00010	124.80	0.99103
HPN	121.12	0.00556	108.52	0.89797	0.00007	118.48	0.94636
PSS	591.38	0.00575	581.16	0.99889	0.00001	635.87	0.99254
PSP	104.98	0.00519	109.04	0.97303	0.00006	120.43	0.93639
Spherical PP	27.17	0.02456	26.44	0.99748	0.00550	26.68	0.99439
Spherical PS	1777.14	0.01471	1737.21	0.99837	0.00002	1784.51	0.99680
Spherical PE	185.01	0.00599	179.18	0.99512	0.00004	193.22	0.99378
PPJ-salinity 20 ‰	36.60	0.00421	36.42	0.91666	0.00011	41.80	0.89246
PPC-salinity 20 ‰	287.10	0.00586	274.91	0.82117	0.00003	293.40	0.87515
HPZ-salinity 20 ‰	32.90	0.01361	32.92	0.99794	0.00098	34.00	0.99876
HPN-salinity 20 ‰	142.40	0.01759	137.35	0.98628	0.00032	142.10	0.99616
PSS-salinity 20 ‰	628.40	0.01381	503.41	0.80022	0.00004	537.70	0.84307
PSP-salinity 20 ‰	39.00	0.00408	38.55	0.72245	0.00013	42.30	0.76810

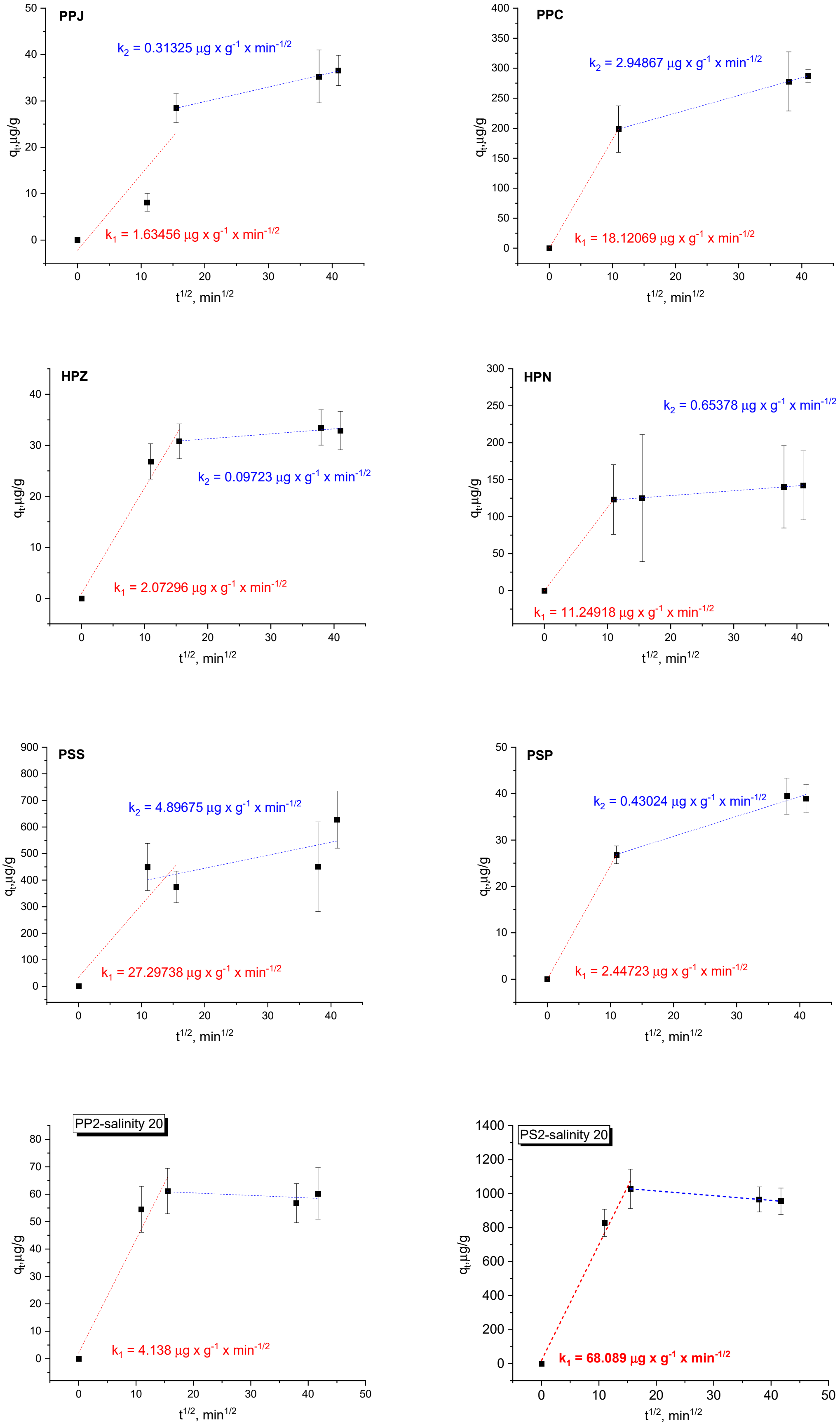
PPJ
k_2 = 0.31325 µg x g^{-1} x $min^{-1/2}$
k_1 = 1.63456 µg x g^{-1} x $min^{-1/2}$
PPC
k_2 = 2.94867 µg x g^{-1} x $min^{-1/2}$
k_1 = 18.12069 µg x g^{-1} x $min^{-1/2}$
HPZ
k_2 = 0.09723 µg x g^{-1} x $min^{-1/2}$
k_1 = 2.07296 µg x g^{-1} x $min^{-1/2}$
HPN
k_2 = 0.65378 µg x g^{-1} x $min^{-1/2}$
k_1 = 11.24918 µg x g^{-1} x $min^{-1/2}$
PSS
k_2 = 4.89675 µg x g^{-1} x $min^{-1/2}$
k_1 = 27.29738 µg x g^{-1} x $min^{-1/2}$
PSP
k_2 = 0.43024 µg x g^{-1} x $min^{-1/2}$
k_1 = 2.44723 µg x g^{-1} x $min^{-1/2}$
PP2-salinity 20
k_1 = 4.138 µg x g^{-1} x $min^{-1/2}$
PS2-salinity 20
k_1 = 68.089 µg x g^{-1} x $min^{-1/2}$
q_t,µg/g
$t^{1/2}$, $min^{1/2}$

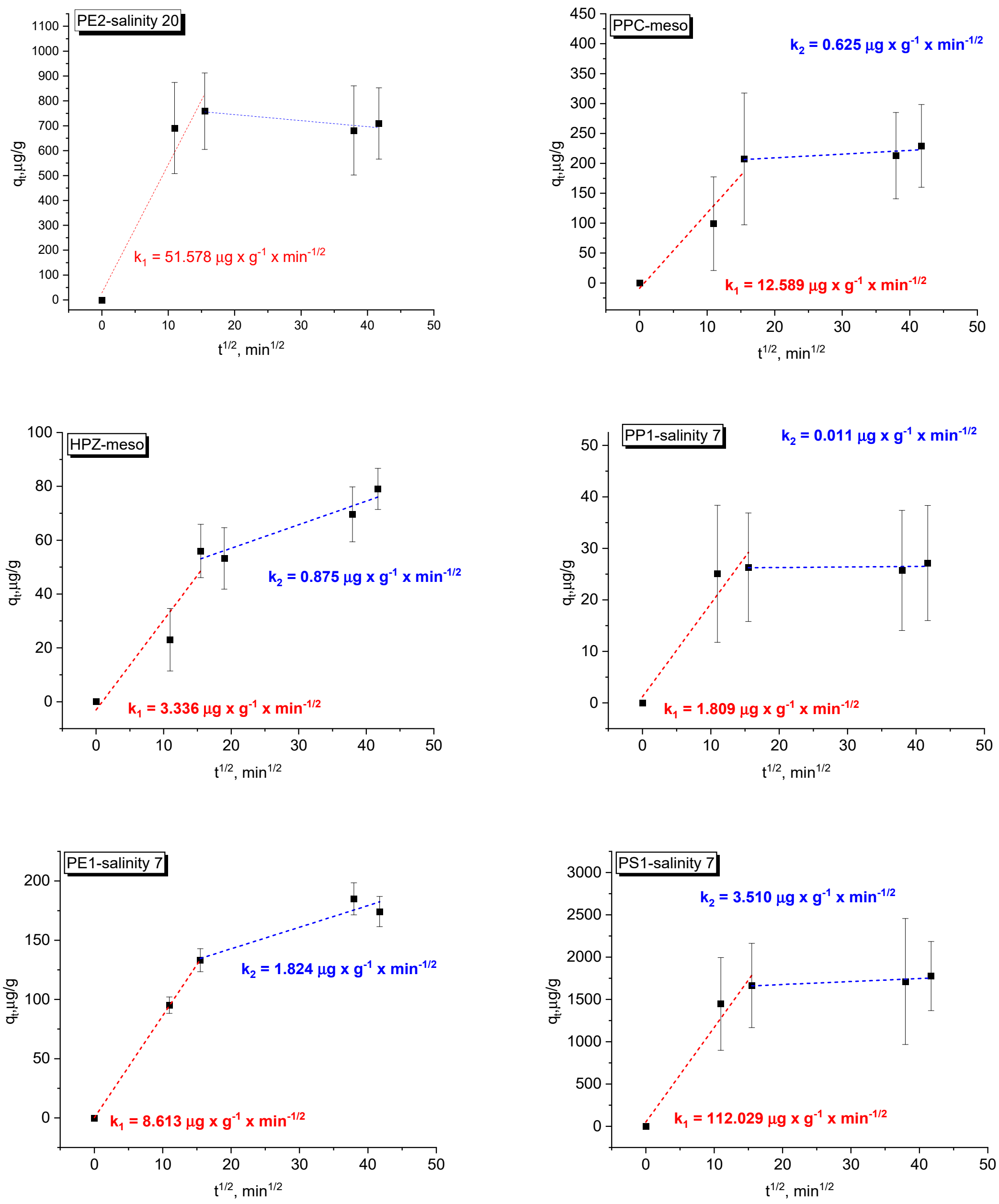

Figure S5. IPD models for all MPs salinity 20‰; secondary mesoplastics; spherical MPs prepared in salinity 7‰ (for PSS mesoplastic k_2 was not calculated).

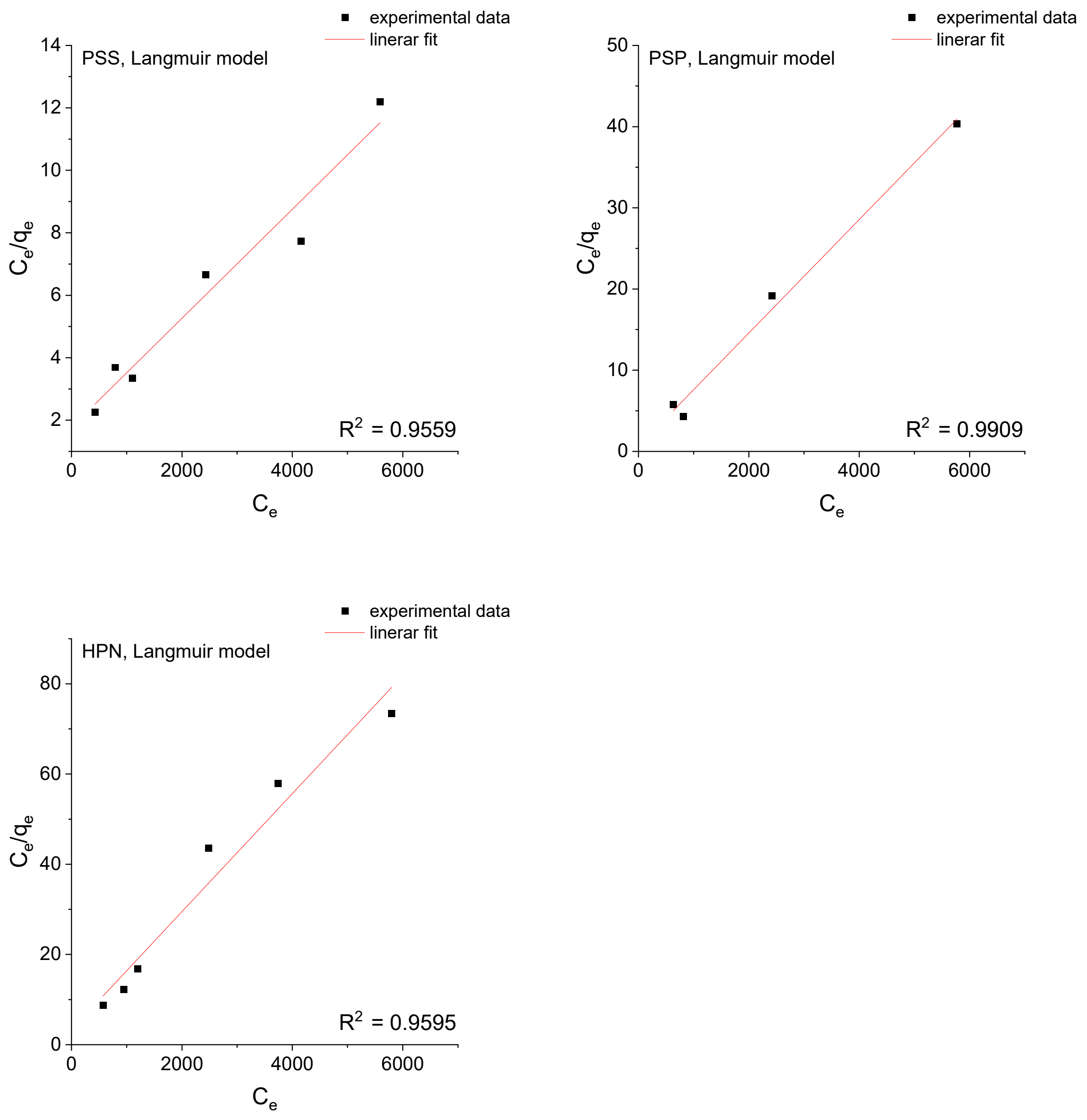

Figure S6. Sorption isotherms for the Langmuir linear model in deionised water (PPC and PPJ are not presented due to a poor fit)

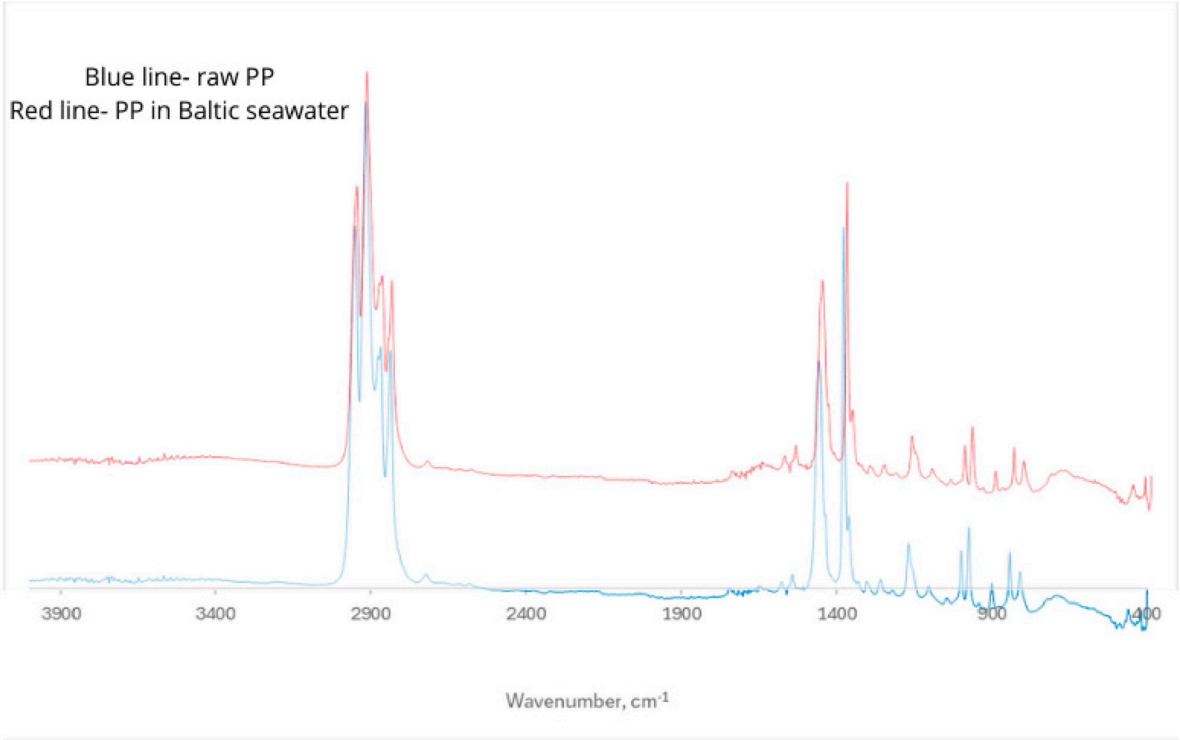

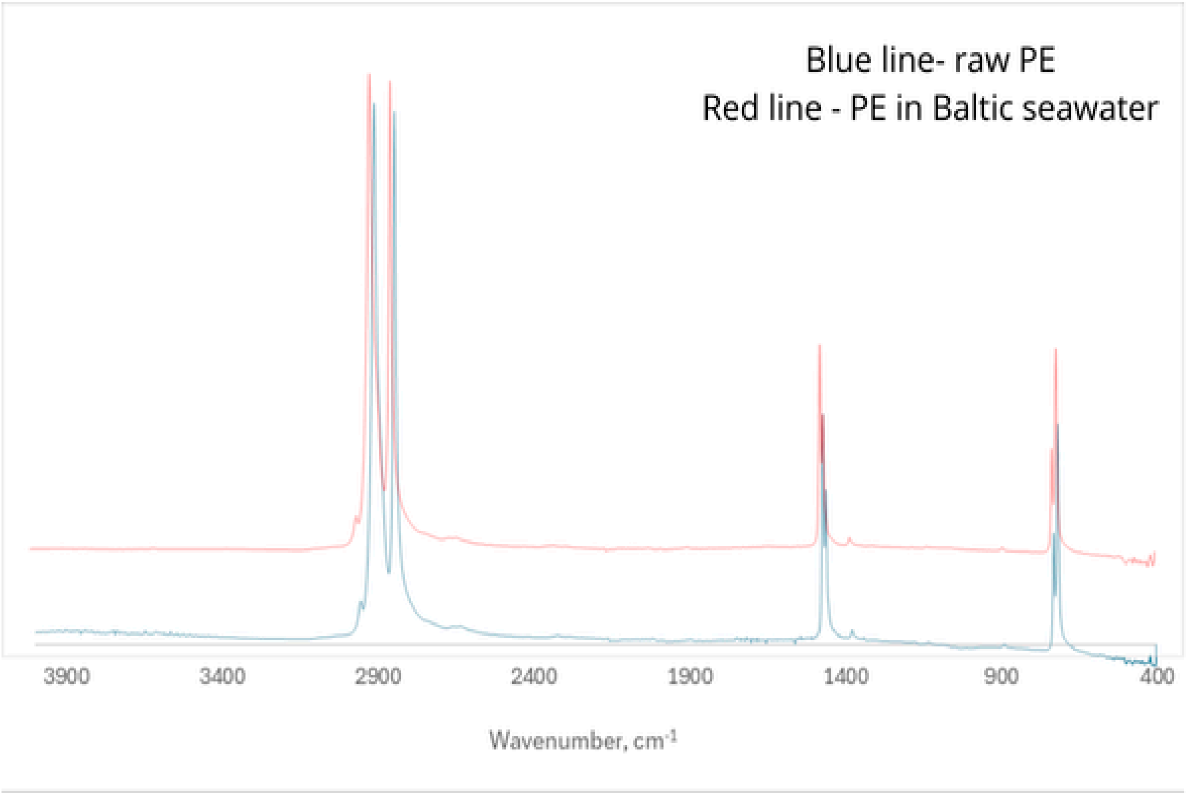

Figure S7. FTIR spectra changes for spherical PP and PE.

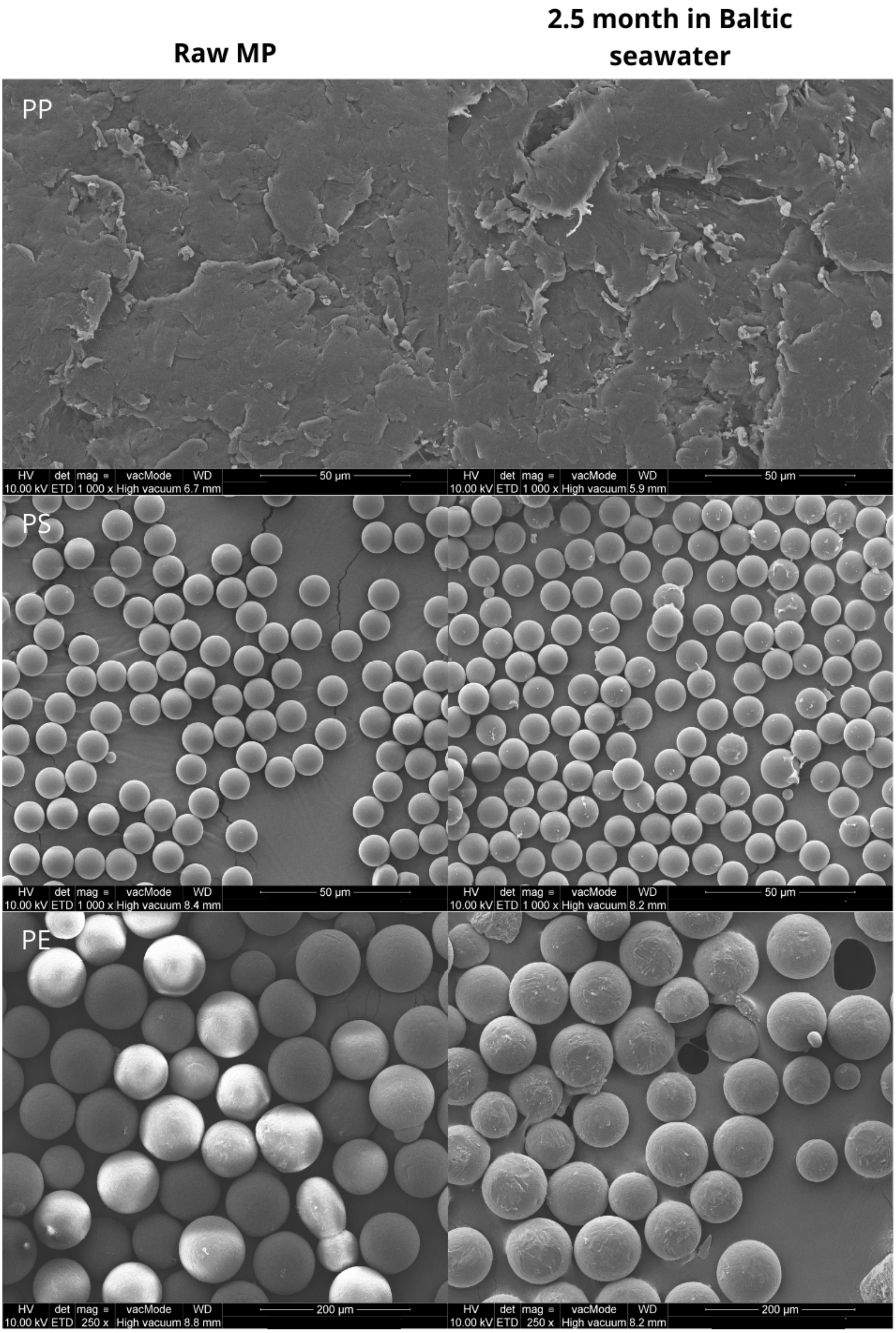

Figure S8. SEM for each spherical MPs, magnification 1000x for PP and PS, 250x for PE.

Table S3. Measured initial conductivity and pH and chosen metal mean ± SD and median in parenthesis.

**Parameters**	**Location: Górki Zachodnie**	**Location: Nowy Port, Gdańsk**	**Location: Gdynia Port**
pH	7.85±0.05	7.71±0.07	7.31±0.12
Conductivity [mS]	10.20	10.90	10.08
Pb [µg/L]	0.70±0.78 (0.41)	1.86±3.21 (0.46)	0.58±0.33 (0.46)
Zn [µg/L]	<2.55	<2.55	<2.55
Cu [µg/L]	<1.22	<1.22	<1.22
Al [µg/L]	<1.85	<1.85	<1.85
Cd [µg/L]	<0.05	<0.05	<0.05
Cr [µg/L]	0.69±0.23 (0.65)	1.02±0.70 (0.77)	0.67±0.24 (0.56)
Se [µg/L]	<1.52	<1.52	<1.52

Table S4. ICP-MS 2030 (Shimadzu, Kyoto, Japan) measurement conditions.

**Parameter and accessories**	**Value**
Radio frequency power generator	1.2 kW
Gas type	argon
Plasma gas flow rate	7.5 L min^{-1}
Auxiliary gas flow rate	1.1 L min^{-1}
Nebulization gas flow rate	0.7 L min^{-1}
Torch	Mini-torch (quartz)
Nebulizer	Coaxial
Spray chamber temperature	3°C
Drain	Gravity fed
Internal Standard	Automatic addition
Sampling depth	7 mm
Collision Cell Gas flow (He)	5.5 ml min^{-1}
Cell Voltage	-21 V
Energy Filter	7.0 V
Number of replicates	3
Integration conditions/number of scans	10

References

[6] Brennecke D, Duarte B, Paiva F, Caçador I, Canning-Clode J, 2016. Microplastics as vector for heavy metal contamination from the marine environment, Estuar. Coast. Shelf Sci. 178, 189-195

[7] Wang X, Zhang R, Li Z, Yana B., 2022. Adsorption properties and influencing factors of Cu(II) on polystyrene and polyethylene terephthalate microplastics in seawater, Sci Total Environ 812, 152573

[15] Godoy V, Blazquez G, Calero M, Quesada L, Martin-Lara MA., 2019. The potential of microplastics as carriers of metals. Environ. Pollut. 255, 113363

[16] Zou J, Liu X, Zhang D, Yuan X., 2020. Adsorption of three bivalent metals by four chemical distinct microplastics, Chemosphere 248, 126

[19] Wang Y, Wang X, Li Y, Li J, Wang F, Xia S, Zhao J., 2020. Biofilm alters tetracycline and copper adsorption behaviors onto polyethylene microplastics, Chem. Eng. J. 392, 123808

[39] Chen CC, Zhu X, Xu H, Chen F, Ma J, Pan K., 2021. Copper Adsorption to Microplastics and Natural Particles in Seawater: A Comparison of Kinetics, Isotherms, and Bioavailability, Environ. Sci. Technol. 55, 13923−13931

[40] Soheilian S, Jordan B, Hatton FL, Hunsicker E, Zhou Z., 2025. Impact of size and UV-ageing of polystyrene nanoparticles on copper(II) adsorption: kinetics and isotherms in aquatic environments, Environ. Sci.: Nano 12, 548

[41] Tang S, Yang X, Zhang T, Qin Y, Cao C, Shi H, Zhao Y., 2022. Adsorption mechanisms of metal ions (Pb, Cd, Cu) onto polyamide 6 microplastics: New insight into environmental risks in comparison with natural media in different water matrices, Gondwana Res, doi: https://doi.org/10.1016/j.gr.2022.06.017

[42] Han X, Wang S, Yu X, Vogt RD, Feng J, Zhai L, Ma W, Zhu L and Lu X., 2021. Kinetics and Size Effects on Adsorption of Cu(II), Cr(III), and Pb(II) Onto Polyethylene, Polypropylene, and Polyethylene Terephthalate Microplastic Particles, Front. Mar. Sci. 8:785146

[50] Liu Q, Wu H, Chen J, Guo B, Zhao X, Lin H, Li W, Zhao X, Lv S, Huang C., 2022. Adsorption mechanism of trace heavy metals on microplastics and simulating their effect on microalgae in river, Environ Res 214, 113777

[76] Ahechti M, Benomar M, El Alami M, Mendiguchía C., 2020. Metal adsorption by microplastics in aquatic environments under controlled conditions: exposure time, pH and salinity, Int. J. Environ. Anal. Chem., DOI: 10.1080/03067319.2020.1733546

[77] Holmes LA, Turner A, Thompson RC., 2014. Interactions between trace metals and plastic production pellets under estuarine conditions, Mar. Chem. 167, 25–32
